# MaMAPK3-MaICE1-MaPOD P7 pathway, a positive regulator of cold tolerance in banana

**DOI:** 10.1186/s12870-021-02868-z

**Published:** 2021-02-17

**Authors:** Jie Gao, Tongxin Dou, Weidi He, Ou Sheng, Fangcheng Bi, Guiming Deng, Huijun Gao, Tao Dong, Chunyu Li, Sheng Zhang, Ganjun Yi, Chunhua Hu, Qiaosong Yang

**Affiliations:** 1 Institute of Fruit Tree Research, Guangdong Academy of Agricultural Sciences; Key Laboratory of South Subtropical Fruit Biology and Genetic Resource Utilization, Ministry of Agriculture and Rural Affairs, Guangdong Key Laboratory of Tropical and Subtropical Fruit Tree Research, Guangzhou, 510640 China; 2 Guangdong Key Laboratory of Ornamental Plant Germplasm Innovation and Utilization, Environmental Horticulture Research Institute, Guangdong Academy of Agricultural Sciences, Guangzhou, 510640 China; 3 Institute of Biotechnology, Cornell University, Ithaca, NY 14853 USA

**Keywords:** *MaMAPK3*, *MaICE1*, *MaPOD P7*, Antioxidant capacity, Cold tolerance

## Abstract

**Background:**

Banana is a tropical fruit with a high economic impact worldwide. Cold stress greatly affects the development and production of banana.

**Results:**

In the present study, we investigated the functions of *MaMAPK3* and *MaICE1* involved in cold tolerance of banana. The effect of RNAi of *MaMAPK3* on Dajiao (*Musa* spp. ‘Dajiao’; ABB Group) cold tolerance was evaluated. The leaves of the *MaMAPK3* RNAi transgenic plants showed wilting and severe necrotic symptoms, while the wide-type (WT) plants remained normal after cold exposure. RNAi of *MaMAPK3* significantly changed the expressions of the cold-responsive genes, and the oxidoreductase activity was significantly changed in WT plants, while no changes in transgenic plants were observed. MaICE1 interacted with MaMAPK3, and the expression level of *MaICE1* was significantly decreased in *MaMAPK3* RNAi transgenic plants. Over-expression of *MaICE1* in Cavendish banana (*Musa* spp. AAA group) indicated that the cold resistance of transgenic plants was superior to that of the WT plants. The *POD P7* gene was significantly up-regulated in *MaICE1*-overexpressing transgenic plants compared with WT plants, and the POD P7 was proved to interact with MaICE1.

**Conclusions:**

Taken together, our work provided new and solid evidence that MaMAPK3-MaICE1-MaPOD P7 pathway positively improved the cold tolerance in monocotyledon banana, shedding light on molecular breeding for the cold-tolerant banana or other agricultural species.

**Supplementary Information:**

The online version contains supplementary material available at 10.1186/s12870-021-02868-z.

## Background

Bananas (*Musa* spp.), including dessert and cooking types, are large herbaceous plants that are perennial but monocarpic [1]. The Musa originated in Southeast Asia and the Western Pacific region, and the domestication process started about 7000 years ago [2]. It involves hybridizations between diverse species and subspecies fostered by human migrations [3], selection of seedless diploid and triploid, and parthenocarpic hybrids widely dispersed by vegetative propagation. Half of the current production relies on some clones derived from a single triploid genotype (Cavendish) [4]. As a staple food and fruit for millions of people, bananas are one of the major export commodities of several developing countries, representing the largest international trade in fruits [5–7].

As an important and restricting factor, temperature determines the development and output of banana, since its growth and production will be irreversibly affected when the temperature is lower than 12 °C [8]. Like many important crops originated in the tropics and subtropics [9], *Musa* spp. appears to lack the mechanism of cold acclimatization, while different cultivars exhibit various cold resistances, in which ‘Dajiao’ (*Musa* spp. ‘Dajiao’; ABB Group) has a stronger cold tolerance compared with Cavendish Banana (*Musa* spp. Cavendish; AAA Group). It is fundamentally necessary to understand the regulatory mechanisms of cold-signaling pathways in banana to improve the cold tolerance in banana cultivars. Due to the highly heterozygous genotype and complex genetic background in molecular biology studies, such as gene function verification related to important traits, the study on cold resistance of bananas has been a challenge for a long time [1, 2].

MAPK cascades have been confirmed in response to many abiotic factors [10–15]. Plant plasma membrane receptors perceive threats and activate mitogen-activated protein kinase kinase kinase (MAPKKK). MAPKKK then phosphorylates mitogen-activated protein kinase kinase (MAPKK), and subsequently, mitogen-activated protein kinase (MAPK) will be phosphorylated by the activated MAPKK [16]. MAPKKK1-MAPKK2-MAPK4/6 cascade has been confirmed to participate in the positive regulation of cold treatment [15, 17]. Over-expression of MAPKK2 in *Arabidopsis thaliana* results in an enhanced cold tolerance, while *mapkk2* mutant exhibits an increased freezing sensitivity [15]. MAPK4/6 is phosphorylated by the activated MAPKK2 for the regulation of downstream components to adapt to the cold stress [15, 18]. A previous report has shown that MYB15 is a phosphorylated substrate of MAPK6, and it is involved in cold resistance [19]. However, a conflict finding has also been reported last year that MPK6 is not the downstream element of MAPKK2, and the MAPKKK1-MAPKK2-MAPK4 pathway constitutively inhibits the activities of MAPK3 and MAPK6 to strengthen the cold resistance [20]. Another cascade, MAPKK4/5-MAPK3/6, can also respond to cold stress. However, there is a controversy about the function of MAPK3/6 in cold resistance. In tobacco and rice, over-expression of *MAPK3* gene confers cold tolerance by stimulating the expressions of *COR* genes [21, 22]. In *Arabidopsis thaliana*, the stability and transcriptional activity of ICE1, a basic-helix-loop-helix transcription factor (bHLH TF) regulating the expressions of *CBF* genes, is reduced by the phosphorylation of MAPK3 and MAPK6, resulting in an impaired cold resistance [20, 23]. Collectively, MAPKKK1-MAPKK2-MAPK4/6 and MAPKK4/5-MAPK3/6 cascades are activated upon cold stress, while their underlying regulatory mechanisms seem to be diverse in different species.

Some bHLH family members have been found to play roles in downstream of MAPKs. Their stability and transcriptional activity are impaired by the phosphorylation of MAPK cascades [20, 21, 23]. In *Arabidopsis thaliana*, MAPKK4/5-MAPK3/6 cascade can negatively modulate the cold response by regulating the stability of ICE1 protein, while the MAPKKK1-MAPKK2-MAPK4 cascade constitutively inhibits the activities of MAPK3 and MAPK6, playing a vigorous role in the cold response [20, 23]. In rice, OsbHLH002/OsICE1 is phosphorylated by OsMAPK3, and the interaction inhibits the ubiquitination of OsbHLH002/OsICE1 to promote the expression of *OsTPP1* and improve the cold resistance [21]. A total of 162, 167, and 152 *bHLH* genes have been identified in *Arabidopsis thaliana*, rice [24], and tomato [25], respectively. Moreover, bHLH proteins in plants participate in a wide variety of biological activities, including flowering [26], trichome and root hair differentiation [27, 28], flavonoid biosynthesis [29], chloroplast growth [30], photomorphogenesis [31], isoquinoline alkaloid [32] and anthocyanin biosynthesis [33]. A great deal of evidence has shown that bHLH transcript factors (*TFs)* also have fundamental functions in response to cold exposure in plants. For example, the bHLH TFs ICE1 and ICE2 of *Arabidopsis thaliana* and SlICE1a of tomato have been shown to participate in the response to cold stress [34, 35]. Because of the huge damage induced by cold exposure, the functions of bHLH TFs in cold resistance are always a hot research topic in fruit science. In apple, MdCIbHLH1 protein has been found to bind to the promoter of *MdCBF2* and positively regulate the cold resistance in different species [36]. In *Poncirus trifoliate*, a bHLH TF named PtrbHLH modulates peroxidase-mediated scavenging of hydrogen peroxide, leading to enhanced cold resistance in transgenic tobacco and lemon [37], and *PtrICE1* from trifoliate orange positively regulates cold resistance of tobacco and lemon by regulating polyamine contents via interplay with arginine decarboxylase [38]. In grape, *VabHLH1* is cloned from a cold-tolerant Chinese wild *Vitis amurensis*, conferring cold tolerance in *Arabidopsis thaliana* [39].

Our laboratory has been undertaking a study on molecular mechanisms of cold tolerance in banana since 2012 using a variety of biology approaches, including transcriptomics, proteomics, phosphoproteomics, and genetic transformation methods [40–44]. We have shown that MAPK cascades, ICE1 signaling pathway and antioxidation mechanism play critical roles in cold resistance of banana. To provide more valuable insights into the correlation between MAPK cascades and ICE1 signaling pathway, we investigated the roles of *MaMAPK3* and *MaICE1* in low-temperature signaling in banana. In the present study, we analyzed the differences of phenotype, gene expression, potential interactions, and antioxidant ability in wide-type (WT), two individual *MaMAPK3* RNAi transgenic ‘Dajiao’ lines and two individual *MaICE*-overexpressing transgenic Cavendish banana lines. Our data showed that MaMAPK3-MaICE1-MaPOD P7 pathway appeared to positively regulate the cold resistance in banana.

## Results

### RNAi of *MaMAPK3* decreases the cold tolerance of transgenic plants

We identified 20 individual equivalent transcripts in Musa genome using the sequences of all *Arabidopsis thaliana MAPK* genes as a reference (Fig. S1A). The expressions of all *MaMAPKs* in Musa genome were determined by quantitative real-time PCR (qRT-PCR) after 3 h of cold stress. Our data indicated that the expression of only *MaMAPK3a* was dramatically increased in Cavendish banana and ‘Dajiao’ upon cold stress (Fig. S1B). Furthermore, the expression of *MaMAPK3a* in ‘Dajiao’ was almost five times higher compared with Cavendish banana. As a result, *MaMAPK3a* (*MaMAPK3* as follows) was chosen as the target in this study.

In the present study, we assessed the subcellular localization of MaMAPK3 in banana protoplast according to the fluorescence of green fluorescent protein (GFP). The open reading frame (ORF) of *MaMAPK3* was in frame with the GFP N-terminus and C-terminus, and no signal was detected in the MaMAPK3-GFP fusion construct. The green fluorescence was detected in both the nucleus and cytoplasm of the cells transformed with the GFP-MaMAPK3 fusion construct (Fig. S2A), and the same result was found in the cells transformed with the control vector (Fig. S2B). These findings suggested that MaMAPK3 was located in both the nucleus and cytoplasm.

We tried to obtain *MaMAPK3* over-expressing line and *MaMAPK3-*RNAi line of ‘Dajiao’ plants. However, all *MaMAPK3* over-expressing plants showed browning and death during germination as well as plant regeneration of resistant embryos (Fig. S3). Therefore, only transgenic ‘Dajiao’ plants with reduced expression of *MaMAPK3* by RNAi approach could be practically used and reported in the present study. A total of 30 resistant RNAi lines (named from MRi-1 to MRi-30) were obtained. *HPT* (1025 bp) and sense strand (200 bp) were amplified from 10 (MRi-2, MRi-4, MRi-5, MRi-13, MRi-14, MRi-15, MRi-19, MRi-22, MRi-24 and MRi-28) of 30 lines (Fig. S4). Southern blotting analysis showed a single copy in MRi-14, MRi-15 and MRi-22 lines (Fig. S5).

**Fig. 1:**
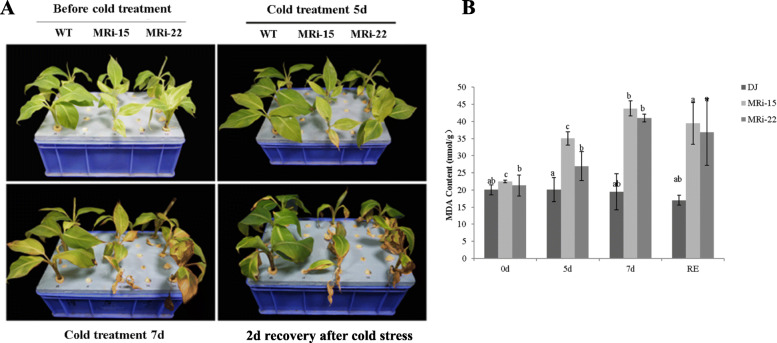
(A) Phenotypes of transgenic ‘Dajiao’ plants (MRi-15 and MRi-22) and wild type (WT) subjected to cold treatment (10 °C for 5 and 7 days), followed by recovery at the normal growth temperature for 2 days. (B) MDA content in WT and transgenic Dajiao (RNAi-22 and RNAi-15) before and after chilling treatment. Data represent the means ± SE of at least three replicates. Letters indicate significant differences from 0 h chilling treatment according to the Student–Newman–Keuls test (*P* < 0.05). (RE means recovery for 2 days)

**Fig. 2:**
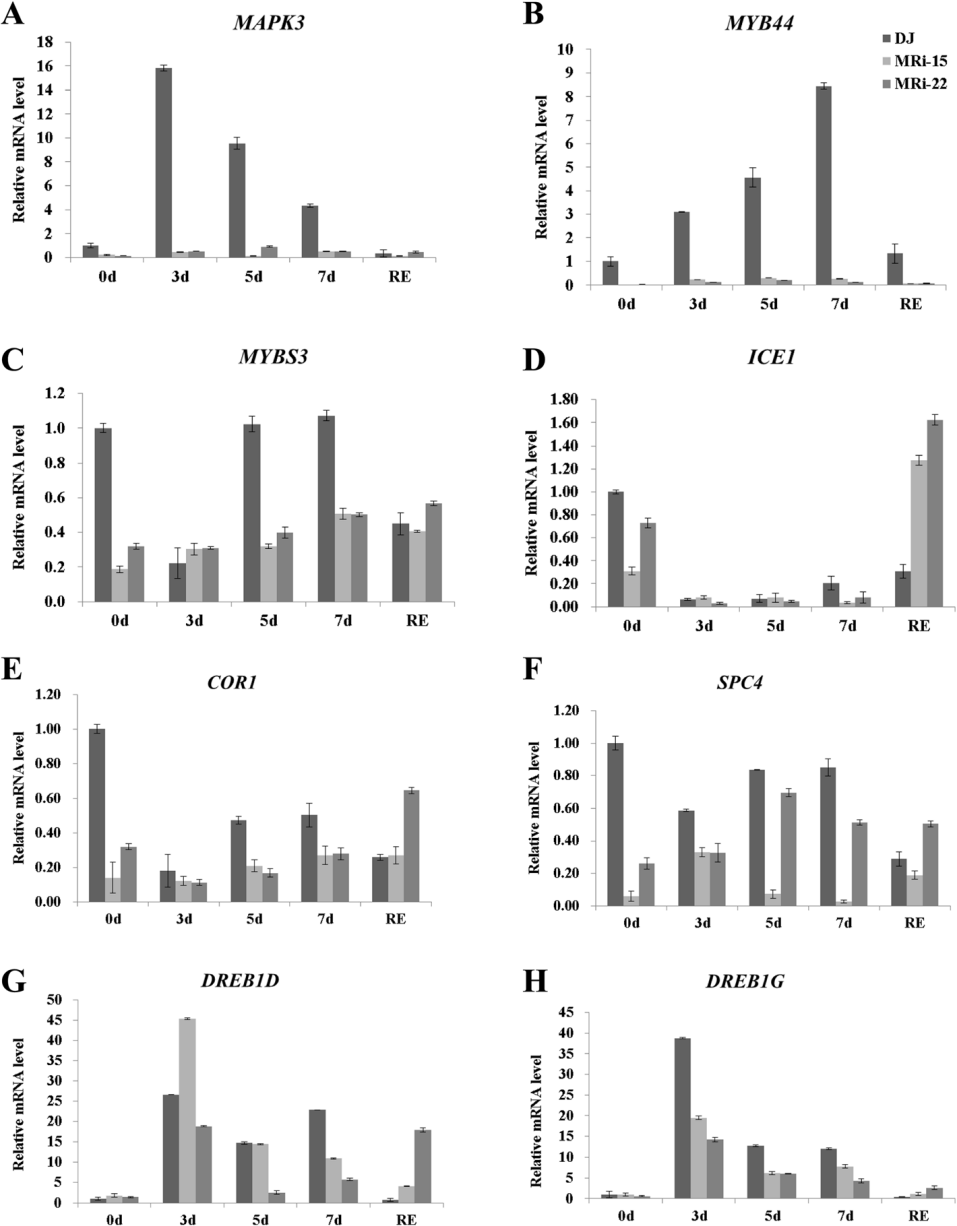
Analysis of relative expression levels of MAPK3 and cold-relative genes by quantitative real-time PCR in wild type and transgenic ‘Dajiao’ lines (MRi-15 and MRi-22) before and after cold treatment. Data represent the means ± SE of at least three replicates. Letters indicate significant differences from 0 h cold treatment according to the Student–Newman–Keuls test (P < 0.05) (RE means recovery for 2 days)

MRi-15 and MRi-22 plants were used for the evaluation of cold resistance. After 5 days of cold stress, ‘Dajiao’ WT showed a normal phenotype, while the leaves of the two transgenic lines became yellow and showed symptoms of water loss. After 7 days of cold stress, minor injuries were found in ‘Dajiao’ WT leaves, while the leaves of ‘Dajiao’ transgenic plants displayed symptoms of severe necrosis and wilting (Fig. 1a). Under most stress conditions, malondialdehyde (MDA) is one of the most representative markers for membrane destruction after free-radical chain reactions [45]. The level of MDA was assessed to investigate the physiological mechanism underlying the decreased cold tolerance of ‘Dajiao’ transgenic plants. The MDA content of ‘Dajiao’ WT plants remained stable with the increased duration of cold exposure. In contrast, the MDA content of two ‘Dajiao’ transgenic lines was significantly increased after cold stress, and such elevation still remained high after recovery for 2 days (Fig. 1b). These results suggested that the suppression of *MaMAPK3* negatively regulated the cold resistance of ‘Dajiao’ plants. The decreased cold resistance in ‘Dajiao’ transgenic plants indicated that the pathways associated with cold resistance might be negatively regulated. To test this conjecture, the expressions of *MaMAPK3* and cold resistance-related genes (including *MYB44*, *ICE1*, *MYBS3*, *DREB1G*, *DREB1D*, *COR1* and *SPC4*) were verified by qRT-PCR under cold stress (10 °C exposure for 0, 3, 5 and 7 days, followed by recovery for 2 days). The expression of *MaMAPK3* was markedly increased in ‘Dajiao’ WT after 3 days of cold stress, while the expression of *MaMAPK3* remained unchanged in ‘Dajiao’ transgenic plants under cold stress (Fig. 2a). Interestingly, the expressions of *MYB44*, *MYBS3*, *ICE1*, *COR1* and *SPC4* in ‘Dajiao’ transgenic plants were suppressed to various degrees compared with the ‘Dajiao’ WT plants (Fig. 2b-f). The expressions of *DREB1G* and *DREB1D* in ‘Dajiao’ transgenic plants were not affected under the normal conditions. However, the expression of *DREB1G* in ‘Dajiao’ WT plants was dramatically increased compared with the ‘Dajiao’ transgenic plants under cold stress (Fig. 2g and h).

### MaMAPK3 interacts with MaICE1

**Fig. 3:**
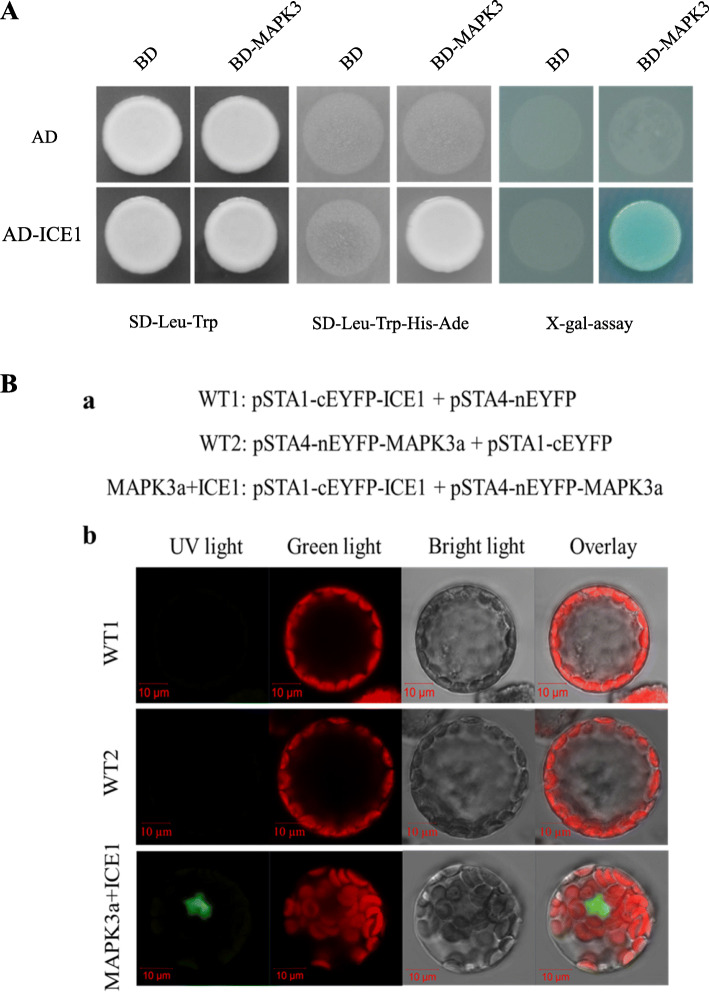
(A) Physical interactions between MaICE1 and MaMAPK3 using Y2H Assays. (The sequences of MaICE1 and MaMAPK3 were cloned from ‘Dajiao’) (B) Biomolecular fluorescence complementation visualization of the interaction between MaMAPK3 and MaICE1. (B-a) Schematic diagrams of the constructs used for biomolecular fluorescence complementation assay. The WT1 and WT2 were two negative controls, respectively. (B-b) The images indicate an interaction between MaMAPK3 and MaICE1 by biomolecular fluorescence complementation

Since the expression of *MaICE1* is suppressed in *MaMAPK3* RNAi ‘Dajiao’ transgenic plants and the functions of *ICE1* are affected by MAPK3 in *Arabidopsis thaliana* and rice [21, 23], we carried out a yeast two-hybrid (Y2H) assay to explore the potential relationship between MaICE1 and MaMAPK3. Strong self-activation activity was detected from the full-length MaICE1 protein when binding to the pGBKT7 vector, whereas AD-MaICE1 (MaICE1 cloned into pGADT7) showed no self-activation activity. Therefore, we chose MaICE1 and cloned it into the pGADT7 vector in our assay. The result of the Y2H assay demonstrated the interaction of MaMAPK3 and MaICE1 (Fig. 3a). We performed bimolecular fluorescence complementation (BiFC) assays to further explore the interplay between MaICE1 and MaMAPK3 in vivo. The results indicated that the fluorescence signal was not detected in cEYFP-*MaICE1*/nEYFP and nEYFP-*MaMAPK3*/cEYFP (the negative combinations), while co-expression of cEYFP-*MaICE1* and nEYFP-*MaMAPK3* yielded strong signals in the nucleus (Fig. 3b). Such fluorescence detection indicated a direct protein-protein interaction between MaICE1 and MaMAPK3, which further supported our Y2H results. The function of *MaICE1* was further investigated and discussed in subsequent experiments.

### Overexpression of *MaICE1* increases the cold tolerance of transgenic plants

The full-length cDNA encoding *MaICE1* (GenBank accession no. KM379133) was isolated from ‘Dajiao’. The subcellular localization of MaICE1 was investigated in banana protoplast according to GFP fluorescence. The ORF of *MaICE1* was in frame with the GFP N-terminus and C-terminus, and no signal was detected in the MaICE1-GFP fusion protein. The GFP-MaICE1 fusion protein was located in the nucleus, whereas the GFP protein in the control was detected in both the cytoplasm and nucleus (Fig. S6).

**Fig. 4:**
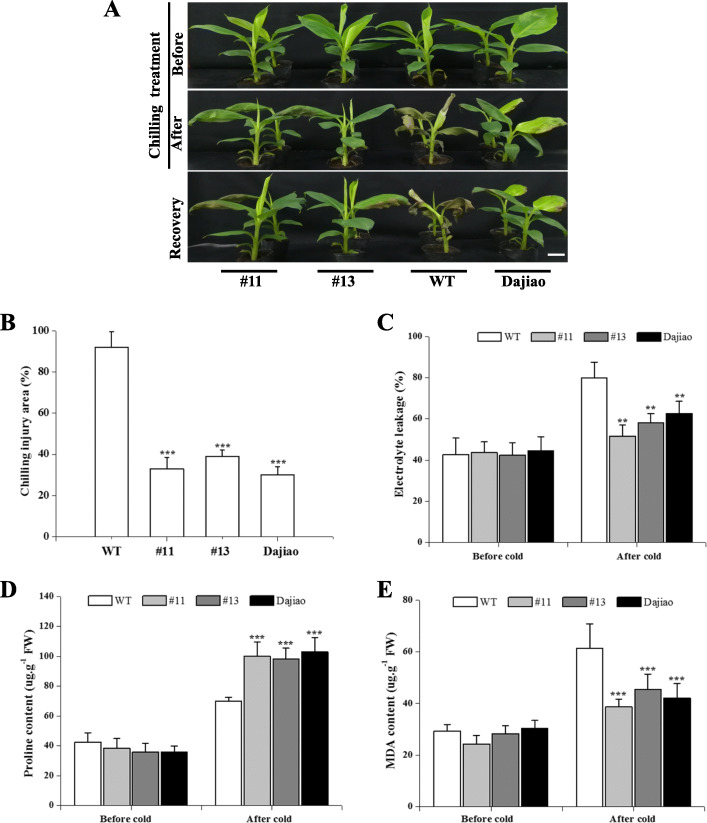
Cold tolerance assay of transgenic Cavendish banana plants. (A) Phenotypes of transgenic Cavendish banana plants (#11 and #13), 'Dajiao' and the wild-type (WT) before and after cold treatment (10 °C for 48 h), followed by recovery in an ambient environment for 3 days (B) Injured area of WT banana and transgenic plants analyzed after cold treatment (bar: 3.5 cm). Effect of *MaICE1* expression in banana on levels of (C) electrolyte leakage, (D) pro and (E) MDA. Two-month-old banana plants of WT, ‘Dajiao’ and transgenic Cavendish banana plants (#11 and #13) were exposed to 10 °C for 48 h. Leaves were collected before and after cold treatment. FW, fresh weight. Each value represents the means of three biological replicates, and vertical bars indicate the SE. **P < 0.01. ***P < 0.001

A total of 13 resistant *MaICE1*-overexpressing lines were obtained. *HPT* (1025 bp) and *ICE1* (200 bp) were amplified from 11 of 13 lines (Fig. S7A and B). Two individual over-expression Cavendish banana lines (#11 and #13) showing a single copy (Fig. S7C) and a high level of *MaICE1* expression (Fig. S7D) were used for cold treatment analysis, along with Cavendish banana WT and ‘Dajiao’ WT plants. Cavendish banana WT, ‘Dajiao’ WT and 2-month-old *MaICE1*-overexpressing transgenic lines (#11 and #13) were exposed to a temperature of 10 °C for 48 h, followed by recovery at normal temperatures. Under normal conditions, the phenotype of transgenic plants could be distinguished between the ‘Dajiao’ WT and Cavendish banana WT plants. When the plants were exposed to 10 °C for 24 h, the leaves of Cavendish banana WT plants showed severe cold injury, whereas only minor injury was observed in the transgenic and ‘Dajiao’ WT plants (Fig. 4a). After 3 days of recovery under the normal condition, the cold injury area of #11 (35.7%), #13 (38.9%) and ‘Dajiao’ WT (32.6%) plants was dramatically improved compared with the WT plants (93.2%) (Fig. 4b).

MDA level, proline (Pro) content, and electrolyte leakage were analyzed to investigate the physiological and biochemical mechanisms underlying the improved cold tolerance of *MaICE1* over-expressing banana plants. Figure 4c shows that after the cold stress, the electrolyte leakage of ‘Dajiao’ WT and transgenic plants was markedly decreased compared with the Cavendish banana WT plants. After exposure to 10 °C for 2 days, all examined plants showed increased levels of Pro. Nevertheless, the content of Pro in transgenic plants was dramatically greater compared with the Cavendish banana WT plants (Fig. 4d). Figure 4e shows that the accumulation of MDA was remarkably lower in the *MaICE1* transgenic lines and ‘Dajiao’ WT plants compared with the Cavendish banana WT plants at the end of the cold stress. These findings supported that the over-expression of *MaICE1* could improve the cold resistance in Cavendish banana.

As a TF, MaICE1 may regulate a variety of downstream elements that can noticeably improve cold resistance in the Cavendish banana over-expression lines. To verify such a hypothesis, we performed a transcriptomic assay to compare the expression profiles of WT and one over-expression line (#13) before and after cold stress. A total of 222 and 496 genes were up-regulated and down-regulated in #13 compared with the Cavendish banana WT plants without cold treatment, respectively, when a 2-fold change was used as a cutoff threshold (Table S1). However, 526 and 196 genes were up-regulated and down-regulated, respectively, in line #13 compared with the Cavendish banana WT plants after only 1 h of cold exposure (Table S2). After 4 h of cold stress, 1109 up-regulated genes and 540 down-regulated genes were identified in line #13 compared with the Cavendish banana WT plants (Table S3), indicating that the gene expression profile was profoundly altered in the transgenic plants. In these differentially expressed genes (DEGs), many genes involved in cold response showed significant up-regulation in transgenic banana under cold stress, such as SPC4 (Ma03_g02760) and COR2 (Ma06_g36560). To find out the effect of *MaICE1* over-expression on MAPK cascades, the expressions of all identified MAPK cascade genes in the transcriptome were analyzed. The expressions of *MaMAPKK2* and *MaMAPK3* were significantly changed in transgenic plants compared with the WT plants under cold stress (Table S4). In Cavendish banana WT plants, the expression of MaMAPKK2 was down-regulated under cold stress, while its expression in transgenic plants was significantly up-regulated under cold stress. The expression of MaMAPK3 was markedly increased in Cavendish banana WT and transgenic plants after 1 h of cold stress. After 4 h of cold stress, the expression of MaMAPK3 was decreased in Cavendish banana WT plants, while it remained at a high level in transgenic plants (Table S4).

**Fig. 5:**
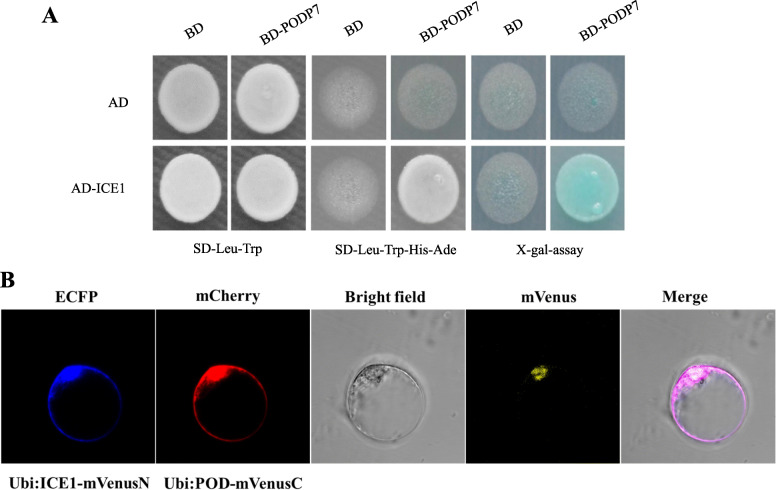
(A) Physical interactions between MaICE1 and MaPOD P7 using Y2H assays. (B) Biomolecular fluorescence complementation visualization of the interaction betweenMaICE1 and MaPOD7. In the BiFC assay, *MaICE1* and *MaPOD7* DNA fragments were fused with the split mVenus N-terminal and C-terminal in the vectors of pRTVnVN and pRTVnVC, which the marker genes ECFP and mCherry were added into the pRTVnVN and pRTVnVC vectors, respectively. After co-transfection, we detected clear yellow fluorescence signals on the nucleus of the transfected protoplasts, indicating the formation of reconstituted mVenus through the MaICE1-MaPOD interaction

Additionally, out of these DGEs, a *Peroxidase P7* (*POD P7*) (ID NO. Ma10_g27800) drew our particular attention. Under the normal condition, the expression of *MaPOD P7* was significantly up-regulated in line #13 compared with Cavendish banana WT plants, while its expression remained at a high expression level during cold treatment. In our previous research, this MaPOD P7 protein is localized in the plasma membrane and chloroplast, which plays a critical role in the cold resistance of ‘Dajiao’ (He et al., 2018). Besides, the interaction between MaICE1 and MaPOD P7 was confirmed by Y2H systems (Fig. 5a) as well as BiFC assays (Fig. 5b).

### Antioxidant capacity analysis in the transgenic plants

**Fig. 6:**
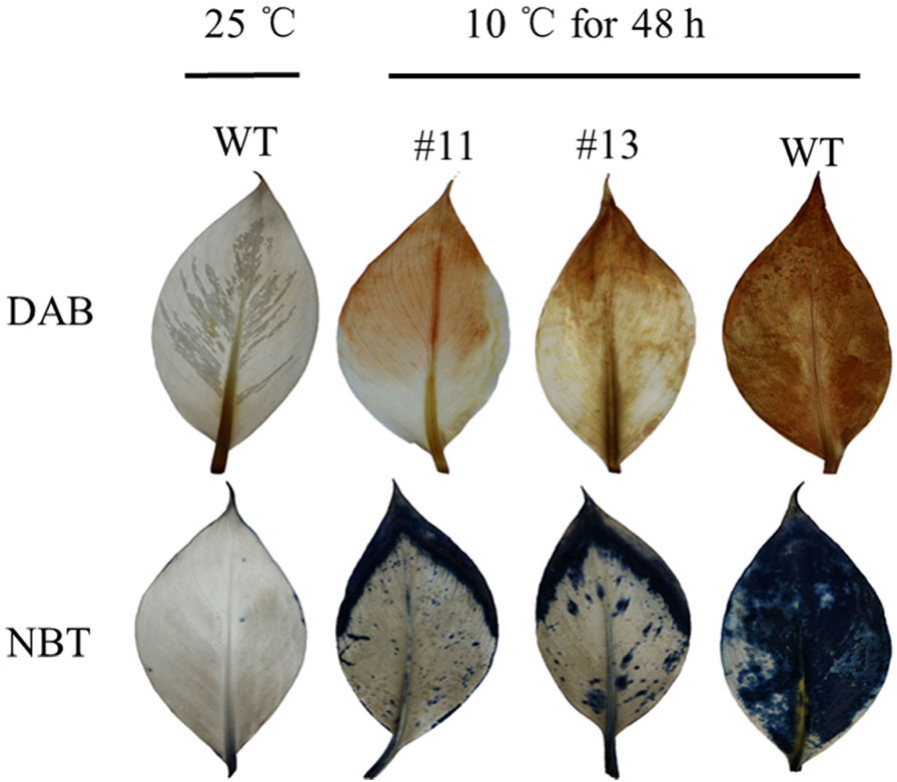
Nitroblue tetrazolium (NBT), and 3,3′-diaminobenzidine (DAB) to analyze the accumulation of O_2_^−^ and H^2^O^2^ in the transgenic Cavendish banana (#11and #13) and wild type plants

*POD P7* is a gene related to antioxidant capacity. To evaluate antioxidant capacity in Cavendish banana WT and *MaICE1*-overexpressing transgenic plants, H_2_O_2_ and O_2_^−^ contents in Cavendish banana WT and *MaICE1*-overexpressing transgenic Cavendish banana leaves were determined by 3,3′-diaminobenzidine (DAB) and Nitroblue Tetrazolium (NBT) staining. Our data indicated that the H_2_O_2_ and O_2_^−^ contents were significantly lower in transgenic Cavendish banana plants compared with Cavendish banana WT plants under cold stress (Fig. 6). According to these findings, we speculated that MaICE1-MaPOD P7 interaction played a fundamental role in cold resistance in *MaICE1*-overexpressing transgenic Cavendish banana.

**Fig. 7:**
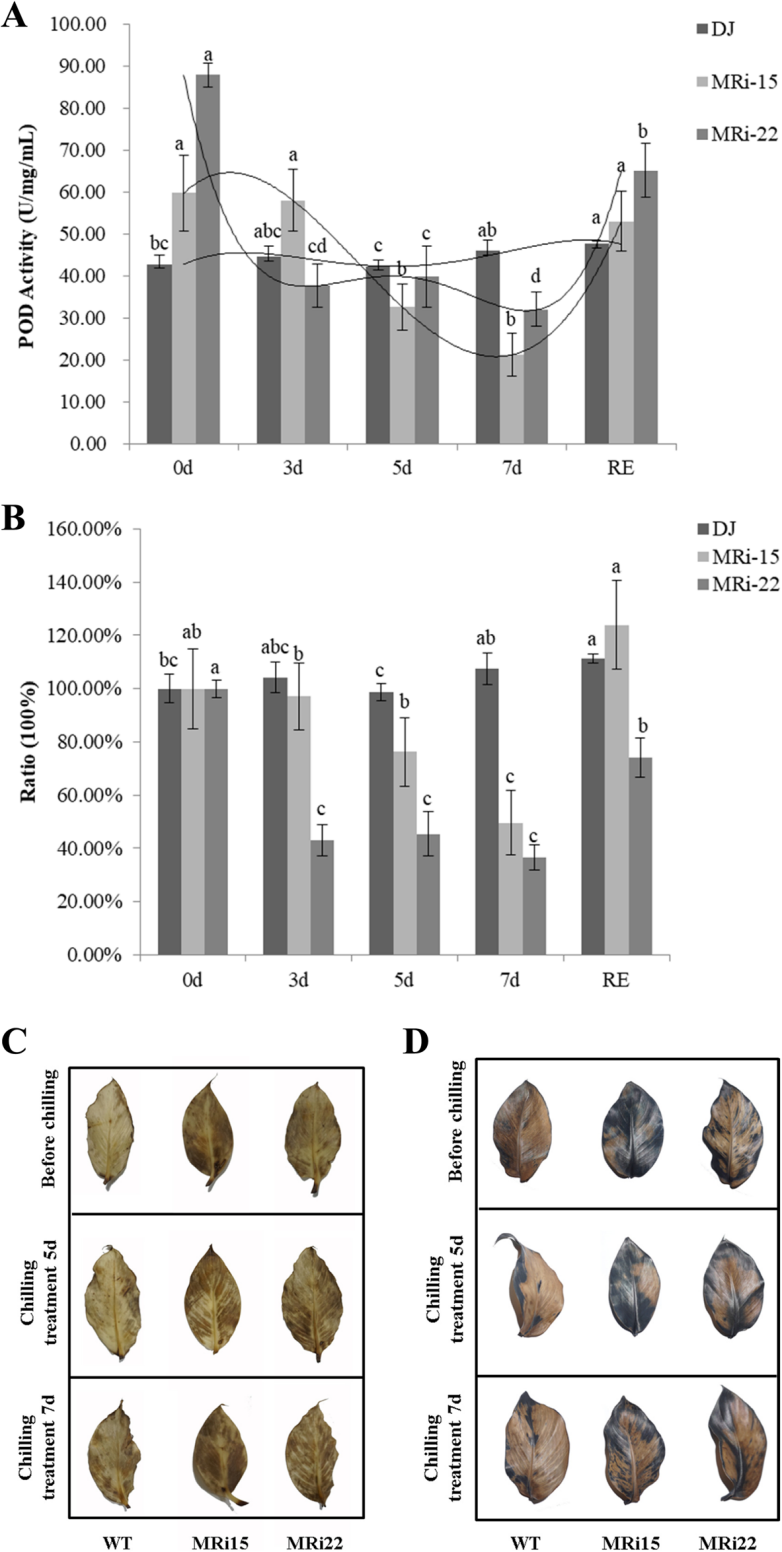
(A) POD activity, (B) the ratio of POD activity,(C) DAB straining, (D) NBT straining to analyze the accumulation of O_2_^−^ and H^2^O^2^ in wild type and transgenic ‘Dajiao’ (RNAi-22 and RNAi-15) before and after cold treatment. Data represent the means ± SE of at least three replicates. Letters indicate significant differences from 0 h cold treatment according to the Student–Newman–Keuls test (P < 0.05) (RE means recovery for 2 days)

Since POD P7 was found to be significantly changed in transgenic Cavendish banana with *MaICE1* over-expression and there was an interaction between MaMAPK3 and MaICE1, we hypothesized that a decreased oxidoreductase activity occurred in *MaMAPK3* RNAi transgenic ‘Dajiao’ plants with the inhibition of *MaMAPK3* by RNAi. The POD activity of ‘Dajiao’ WT and transgenic ‘Dajiao’ plants was examined. Under the normal condition, the POD activity was higher in transgenic ‘Dajiao’ plants compared with ‘Dajiao’ WT plants (Fig. 7a). However, after cold treatment, the POD activity remained stable in ‘Dajiao’ WT plants, while it was significantly decreased in transgenic ‘Dajiao’ plants (Fig. 7b). To figure out the reason why the POD activity was higher in *MaMAPK3* RNAi transgenic ‘Dajiao’ plants, we measured the H_2_O_2_ and O_2_^−^ contents by DAB and NBT staining. We found that the contents of H_2_O_2_ and O_2_^−^ were dramatically greater in transgenic ‘Dajiao’ plants compared with the ‘Dajiao’ WT plants under the normal condition (Fig. 7c and d), which might explain the greater POD activity in transgenic ‘Dajiao’ plants under the normal condition.

## Discussion

Plants have evolved a variety of sophisticated defense mechanisms to deal with different stresses. In plants, complex and rapid signaling pathways are responsible for the response and adaptation to cold stress, and such pathways can impair a wide range of cellular activities, such as physiology or biochemistry changes, transcriptional regulation, cell division and morphogenesis, protein folding and metabolic changes in nutrient flux [46–48]. In our lab, we have investigated the molecular mechanisms of cold resistance in bananas for a long time. Based on the previous results from proteomics, transcriptomics, gene function analysis, membrane proteomics, and phosphoproteomics [40–43, 49], we have found several independent pieces of evidence for mechanisms underlying the cold resistance. In the present study, we aimed to investigate the potential connections for those independent mechanisms.

### MaMAPK3 is a positive regulator of cold tolerance

Transcriptome analysis has indicated that the expression of *MaMAPK3* in ‘Dajiao’ is significantly higher compared with Cavendish banana under cold stress [42]. The pathways mediated by MAPKs not only display an important effect on the development of plants but also participate in regulatory responses to stresses [10, 50, 51]. MAPK family is divided into four subgroups based on sequence and structural similarity, namely A, B, C, and D subgroups [52]. It is well known that MAPKs belonging to A and B subgroups participate in the response to biological and abiotic stresses. In rice, OsMAPK3 belonging to subgroup A acts as a positive regulator by interacting with the phosphorylated OsbHLH002/ICE1 protein [21], while AtMAPK3 has been reported as a negative regulator in cold resistance of *Arabidopsis thaliana* [20, 23]. In our present study, *MaMAPK3* was activated by cold stress, and the phylogenetic tree analysis revealed that MaMAPK3 belonged to subgroup A (Fig. S1). Therefore, we hypothesized that MaMAPK3 participated in the regulation of cold resistance in ‘Dajiao’ that has a stronger cold tolerance than other banana cultivars.

We investigated MaMAPK3 using both knock-in and knock-down (by RNAi) transgenic lines of MaMAPKs. We did not successfully establish the over-expression lines as apparently too much MaMAPK3 protein was likely to disturb the balance of growth. However, the transgenic plants with suppressed expression of *MaMAPK3* showed dwarf features and cold sensitivity compared with the WT plants. Moreover, the phenotype analysis of transgenic plants allowed us to conclude that MaMAPK3 indeed participated in cold response and positively regulated cold resistance in “Dajiao”. To understand the effect of *MaMAPK3* RNAi on the global transcriptional level, we first investigated the expression profiles of seven marker genes [41, 48, 53–56], which have been well known to participate in cold resistance, in the transgenic lines. Our data indicated that the suppression of *MaMAPK3* exerted a negative effect on cold resistance of “Dajiao” at the transcriptional level. Besides, we found that the reduced oxidoreductase activity in transgenic ‘Dajiao’ could further rationalize and connect to the importance of POD function. It should be noted that MAPK3 might play different roles in different species. In *Arabidopsis thaliana*, only AtMAPK4/6 and AtMAPK3 can interact with AtICE1, and AtMAPK3 functions as a negative modulator under cold stress [20, 23], whereas 10 of 22 MAPKs in banana can interact with MaICE1 (Fig. S8). These results indicated that there was a large difference between *Arabidopsis thaliana* and banana in terms of MAPK3-mediated regulatory mechanism.

### MaICE1 is a positive regulator of cold tolerance

The largest TF family in plants is composed of bHLH proteins [54, 57]. A great deal of evidence has shown that bHLH TFs have fundamental functions in plant responses to different abiotic stresses, such as drought stress [58], salinity stress [59], and cold stress [35, 37, 54, 60]. The bHLH proteins consist of conserved bHLH signature domains composed of one basic region at the N-terminal end and one HLH region at the C-terminal end [24, 38]. The specificity of the DNA-protein interactions is determined by the basic region consisting of approximately 15 amino acids, including several basic residues [37]. There are two amphipathic α-helices in the HLH region, which are connected by a loop region of variable length and play a critical role in the formation of homodimers or heterodimers [24]. The basic region allows bHLH TFs to bind to consensus E-box (5′-CANNTG-3′) or G-box (5′-CACGTG-3′) cis elements to regulate the gene expression [25]. As TFs, MYC-type proteins are localized in the nucleus to exert their regulatory functions, and most isolated bHLH proteins are localized in the nucleus [37, 54, 61]. Similarly, MaICE1 was confirmed to be localized in the nucleus after the transformation of banana protoplasts (Fig. S6), suggesting that MaICE1 was a nuclear protein.

For now, the roles of bHLH homologs in ‘Dajiao’, a very cold-hardy plant, remain largely unexplored. Compared with the Cavendish banana, ‘Dajiao’ is significantly cold-tolerant in winter, which can undergo low temperatures down to 0–4 °C [41, 49]. Therefore, it is highly necessary to characterize the functions of bHLH genes from ‘Dajiao’ to clarify the cold signaling pathway associated with cold resistance and identify useful gene candidates for genetic manipulation [41]. We transformed *MaICE1* into a cold-sensitive perennial plant, Cavendish banana with great agronomic value. Constitutive expression of *MaICE1* led to significant changes in the cold resistance of the transgenic plants. Over-expression of *MaICE1* in banana significantly decreased the cold stress-induced damage (Fig. 4a) and resulted in better plant phenotypes compared with the WT plants after cold stress (Fig. 4b). These above-mentioned findings were also accompanied by an elevated level of Pro (Fig. 4d), reduced MDA content (Fig. 4e) and electrolyte leakage (Fig. 4c). Collectively, our results showed that *MaICE1* played a beneficial role in cold resistance. Previous studies have shown that banana plants over-expressing stress-responsive TFs, such as AtCBF1 and MusabZIP5, frequently exhibit growth restriction [41, 44]. However, except that the growth period was 1 month longer than WT plants, no apparent phenotypic changes were caused by *MaICE1* over-expression compared with the WT plants under normal growth conditions, implying that MaICE1 could be potentially used in genetic manipulation to ameliorate cold resistance in banana.

It is well known that as a highly complex process, the stress response is mediated by several signaling pathways [41]. To clarify the molecular mechanisms underlying the improved cold resistance, we compared transcriptional profiles between Cavendish banana WT plants and the over-expression transgenic line (#13) under normal conditions and cold stress. We found that comprehensive transcriptomic modifications were induced in the transgenic line by the over-expression of *MaICE1*. It is worth noting that MaICE1 both promotes and inhibits the expressions of many genes at the mRNA level, suggesting both positive and negative effects on the expression atlas. Such a finding is not an exception since many studies have reported the extensive transcriptional reprogramming in transgenic plants over-expressing a TF compared with their WT controls [37, 62]. In all DEGs found in transcriptomics, the expression of *POD P7* (Ma10_g27800) exhibited the largest difference before and after the cold stress. In our previous quantitative proteomics and membrane proteomics analyses between cold-tolerant ‘Dajiao’ and cold-sensitive Cavendish banana, POD P7 protein is the only peroxidase with an increased abundance localized in the plasma membrane and chloroplast. The increased POD P7 expression appears to be a key cellular adaptation contributing to the cold tolerance of ‘Dajiao’ by involving in decreased lipid peroxidation [49]. Besides, in our new proteomics data (data not shown), the abundances of four SOD and 27 POD proteins were significantly increased in *MaICE1*-overexpressing Cavendish banana plants, and 10 of 27 PODs were POD-P7 (including Ma10_g27800) or POD-P7 like proteins. Moreover, the interaction between MaICE1 and MaPOD P7 was confirmed by Y2H systems and BIFC assays (Fig. 5), indicating that ICE1-POD P7 interactions played a key role in cold tolerance in transgenic banana.

As shown in Supplementary Table 1, 2 and 3, the number of DEGs after 4 h of cold stress (1109 up-regulated and 540 down-regulated genes) was significantly higher compared with the normal conditions (222 and 496, respectively). We speculated that MaICE1 underwent certain undetermined modifications upon exposure to cold stress, leading to the expression or suppression of a set of stress-responsive genes involved in the plant stress-responsive signaling network. For now, it has been reported that the activity of ICE1 can be positively regulated by the phosphorylation of OST1 [63] and negatively mediated by the phosphorylation of MAPK3 [20, 23] in *Arabidopsis thaliana* under cold stress. Based on the Y2H and BiFC assays, MaMAPK3 was found to interact with MaICE1 (Fig. 3). The results indicated that MaMAPK3-MaICE1 played a central role in cold response in banana.

### MaMKK2 interacts with MaMAPK3

MKK2 is an intermediate node in MAPKKK-MAPKK-MAPK cascades. Over-expression of MKK2 affects the expression of the CBF gene by phosphorylation of downstream MPK6/MPK4 to enhance the cold tolerance of *Arabidopsis* [15]. In banana, however, we found that the phosphorylation level of MaMKK2 was significantly increased in cold-resistant ‘Dajiao’, while such elevation was not detected in cold-sensitive banana. These results revealed the important role of MaMKK2 in cold resistance of banana [40]. After we found that MAPK3 RNAi ‘Dajiao’ plants showed a phenotype with decreased cold resistance, the Y2H method was used to assess the interplay between MKK2 and MAPK3, and we found that MaMKK2a could indeed interact with MaMAPK3 (Fig. S11). Therefore, based on the significantly up-regulated expressions of MKK2 and MAPK3 genes in *ICE1* over-expressing transgenic plants, we believed that the interaction between MKK2 and MAPK3 was most likely involved in the cold resistance of banana.

## Conclusions

**Fig. 8:**
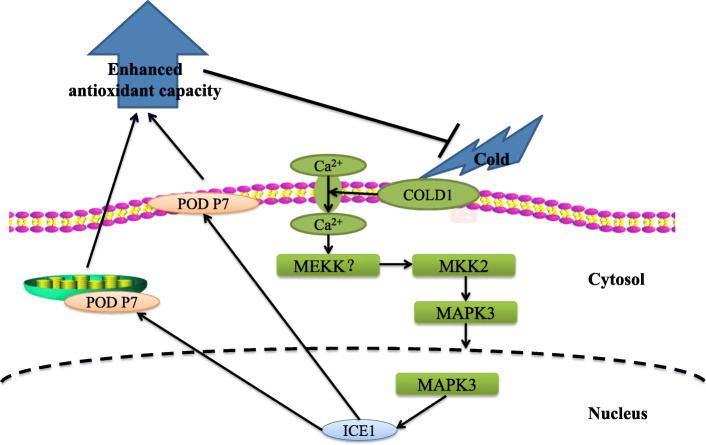
A working model showing the roles of MAPK3-ICE1-POD P7 pathway in ‘Dajiao’ cold stress response

Collectively, the MAPK3-ICE1-POD pathway played a critical role in banana cold resistance. Based on the findings acquired from this study, we proposed a model towards a cold resistance mechanism by the MAPK3-ICE1-POD pathway (Fig. 8). Under cold stress, MEKK?-MKK2-MPK3 cascades were rapidly activated. Subsequently, the ICE1 expression was promoted by the activated MAPK3, thus facilitating the expression of membrane-associated gene *POD P7* gene. The MKK2-MPK3-ICE1-POD P7 pathway positively affected cold response.

## Methods

### Plant materials, growth conditions, and cold treatments

‘Dajiao’ (*Musa* spp. “Dajiao”; ABB group; accession No. LCDJ_01) and Cavendish banana (*Musa* spp. AAA group) were harvested from the National Banana Germplasm Repository, Institute of Fruit Tree Research, Guangdong Academy of Agricultural Sciences. For cold treatment, the potted plantlets were cultivated up to the five-leaf-stage in a growth chamber under the conditions as follows: temperatures of 30/28 °C (day/night), the photon flux density of 240 μmol m^− 2^ s^− 1^, 12-h photoperiod, and relative humidity of 60–80%. Three plants with a uniform growth status were then placed into a chamber set at 10 °C for 0, 1, 3, 6, 24 and 48 h in the dark. The temperature treatment repeated three times in a single growth chamber. The fully expanded leaves on the top of the plants/plantlets were harvested at the indicated time intervals, frozen in liquid nitrogen right away, and preserved at − 80 °C before further analysis. All specimens at each time point were collected in triplicate. Three plantlets constitute a biological for each separate cultivar used in this study.

### Gene isolation and sequence analysis

Total RNA was extracted from ‘Dajiao’ leaves by a plant RNA extraction kit (Code No. 9767, TaKaRa, Dalian, China), and then 1 μg purified RNA was reversely transcribed into cDNA using a PrimeScript RT Reagent Kit according to the manufacturer’s protocol (TaKaRa). According to the cDNA sequence of *MaICE1* obtained from the ‘Dajiao’ transcriptome data [43], partial ‘Dajiao’ *ICE1* cDNA fragments were amplified by PCR using primers harboring *Spe*I and *Bam*H I restriction sites (GSP1, Table S5). The sequence of *MaMAPK3* was acquired from the genome database of Musa (http://banana-genome-hub.southgreen.fr/home) [1]. Sequences were aligned using ClustalW [64], and the phylogenetic tree was constructed using MEGA 7 software [65].

### Sub-cellular localization

The ORF of MaMAPK3 was in frame with the GFP N-terminus and C-terminus, and no signal was detected in the MaMAPK3-GFP fusion construct, the signal of GFP-MaMAPK3 was used. The full-length cDNA of *MaMAPK3* was subcloned into the pMD18-T vector (TaKaRa). Plasmid pMD18-T containing *MaMAPK3* was amplified using primers GSP3 (Table S5) containing *Sal* I and *Cla*I restriction sites to assess the subcellular localization of MaMAPK3. The ORF of MaICE1 was in frame with the GFP N-terminus and C-terminus, and no signal was detected in the MaICE1-GFP fusion construct, the signal of GFP-MaICE1 was used. The full-length cDNA of *MaICE1* was subcloned into the pMD18-T vector (TaKaRa). Plasmid pMD18-T containing *MaICE1* was amplified using primers GSP4 (Table S5) containing *Sal* I and *Cla*I restriction sites to assess the subcellular localization of MaICE1. The PCR product was digested with the above-mentioned enzymes and introduced into the pUC19-GFP vector harboring the GFP reporter gene to generate the fusion construct under the control of the cauliflower mosaic virus 35S promoter (CaMV 35S). The fusion construct and the control vector (pUC19-GFP) were separately introduced into Cavendish banana protoplast as previously described [66]. Images were captured from transiently transformed rice protoplast cells grown at 28 °C using a confocal laser-scanning microscope (LAM510, Carl Zeiss GmbH, Jena, Germany) and analyzed by Image-Pro software.

### Generation of transgenic plants by *Agrobacterium Tumefaciens*-mediated transformation

The full-length cDNA of *MaMAPK3* was subcloned into the pMD18-T vector (TaKaRa, Dalian, China) (Fig. S10A). Plasmid pMD18-T containing *MaMAPK3* was amplified using primers (GSP1) harboring *Spe*I and *Bam*H I restriction sites. The PCR product was digested with these enzymes and introduced into the pCAMBIA 1301-GUS vector (Fig. S10B) to generate the fusion construct 1301-*MaMAPK3*-GUS under the control of the Ubipromoter. The double-stranded RNA interference (dsRNA) construct (Fig. S10C) was generated via a PCR-mediated method using the amplification products from a unique N-terminal region (300 bp) spanning a portion of the 5′-untranslated region and adjacent coding region of the *MaMAPK3* gene. The sense strand was then amplified using a primer combination harboring *Bam*H I and *Hin*d III restriction sites on the opposed ends of the product, whereas the antisense strand was amplified using a primer combination harboring Pst I and Mul I restriction sites on the opposite ends of the product. These two products were introduced into pYL-RNAi under the control of the Ubi promoter. The newly constructed 1301-*MaMAPK3*-GUS plant expression vector and RNAi vector were introduced into *A. tumefaciens* strain EHA105 by heat shock [41]. The over-expression vector was adopted for the transformation of ‘Dajiao’ suspension cultured cells (ECSs), and the RNAi vector was used for the transformation of ‘Dajiao’ ECSs as previously described [41, 67]. Hygromycin-resistant plants were selected and identified by PCR (*HPT* and *MaICE1*) using two pairs of primers (GSP5 and GSP6, Table S5). Only those yielding the expected PCR fragments by both primers were regarded as positive. Moreover, the expression of *MaMAPK3* at the mRNA level was examined by qRT-PCR (primer set GSP6, Table S5). The *MaACT1* gene (primer set GSP7, Table S5) was selected as a housekeeping gene. Positive banana plants were multiplied vegetatively using meristems of in vitro plantlets. The rooted plantlets were hardened in the greenhouse and used for further analyses.

Specific primers (GSP2) containing Spe I or BamH I restriction sites were used to amplify *MaICE1* cDNA. The PCR product was digested with *SpeI* and *Bam*H*I*, and then ligated into binary vector pOx driven by the *Zea mays* L. polyubiquitin promoter. The constructed binary vector was denoted as pOx-*MaICE1*. The newly constructed pOx-*MaICE1* plant expression vector was introduced into *A. tumefaciens* strain EHA105 by heat shock [37]. The over-expression vector was adopted for the transformation of Cavendish banana ECSs as previously described (Dou et al., 2016; Hu et al., 2013). Hygromycin-resistant plants were selected and identified by PCR using two pairs of primers (GSP5and GSP8). Only those yielding the expected PCR fragments by both primers were regarded as positive. Moreover, the expression of *MaICE1* at the mRNA level was examined by qRT-PCR (primer set GSP9, Table S5). The *MaACT1* gene (primer set GSP7) was selected as a housekeeping gene. Positive banana plants were multiplied vegetatively using meristems of in vitro plantlets. The rooted plantlets were hardened in the greenhouse and used for further analyses.

The RNAi construct for *MaMPAK3* gene suppression was amplified using the primers GSP10 and GSP11, then the PCR products were cloned into pYLRNAi, which was kindly provided by Dr. Yao-Guang Liu from College of Life Sciences, South China Agricultural University, China. The following procedure was carried out as previously described.

### Physiological analyses of *MaMAPK3* RNAi transgenic ‘Dajiao’ plants

The first young leaf on the top of each of the three plants was collected at each time point (10 °C for 0, 3, 5, and 7 days and recovery after cold stress for 2 days) for each biological replicate. The MDA content was determined by the plant MDA assay kit (Nanjing Jiancheng Bio, Nanjing, China), and the POD activity was tested by the peroxidase assay kit (Nanjing Jiancheng Bio, Nanjing, China). Five replicates were performed for each sample. Three independent experiments were performed as biological replicates.

### Cold tolerance assays of the *MaICE1* transgenic banana plants

To avoid interference from plant size and reproduction stage, all plants were chosen from 2-month-old hardened transgenic Cavendish banana, Cavendish banana WT and ‘Dajiao’ plants, which were grown in plastic pots filled with a mixture of vermiculite and soil (1:1) under a photoperiod of 16 h/8 h (light/dark) at 25 °C. At this stage, there were no apparent differences in plant size and growth between the transgenic and Cavendish banana WT plants. To assess their cold resistance, transgenic lines and WT plants were directly exposed to 10 °C for 48 h without pre-acclimation, followed by recovery at an ambient environment for 5 days [41, 42]. Cold injury and recovery of the plants were recorded and photographed. The degree of cold damage was assessed after recovery as previously described [41].

Two transgenic lines (#11 and #13), Cavendish banana WT and ‘Dajiao’ plants, which were subjected to 10 °C for 48 h and subsequent recovery at normal conditions, were adopted in the present study. The leaves were collected before and/or after the cold stress to analyze the electrolyte leakage, Pro content, and MDA level. The leaves used for measurements of electrolyte leakage were cut into 1-cm segments and washed three times with ultrapure water. The segments were placed in tubes containing 5 mL of ultrapure water and incubated at 25 °C. After 2 h, the electrical conductivity of the bathing solution (L1) was measured. Then the tubes were incubated at 100 °C for 20 min and subsequently at 25 °C for 1 h, and the electrical conductivity (L2) was measured again. The relative electrolyte leakage was calculated by the formula (L1-L0)/(L2-L0) × 100 (L0, the conductivity of ultrapure water) [53]. MDA level and the Pro content were determined using the commercially available kits (Nanjing Jiancheng Bioengineering Institute, China). Five replicates were performed for each sample. Three independent experiments were performed as biological replicates.

### RNA-Seq analysis

Transcriptional profiling of Cavendish banana WT and MaICE1-overexpressing plants (transgenic line #13) was conducted by RNA-Seq analysis at the Beijing Genomics Institution (BGI). Three biological replicates were adopted for each genotype under normal conditions and cold stress (10 °C for 1 and 4 h). RNA isolation, library construction, and sequencing on the BGISEQ-500 platform of PE 100 with 30 million reads per sample were performed at BGI (www.genomics.org.cn, BGI, Shenzhen, China). Gene expressions were determined using the RSEM software package [68]. To further confirm the reliability of those transcriptomic data, the expressions of four up-regulated genes were examined by qRT-PCR with specific primers (GSP7, GSP8, GSP9, and GSP10) as shown in Table S5. The DEGseq approach was employed to screen DEGs between groups with the criteria of a fold change ≥2 and adjusted *p*-value ≤0.001 as previously described [69]. Gene Ontology (GO) pathway annotation and enrichment analyses were carried out based on the GO database (http://www.geneontology.org/) and the KEGG pathway database (http://www.genome.jp/kegg/), respectively.

### qRT-PCR analysis

Briefly, 1 μg RNA was reversely transcribed into cDNA using ReverTra Ace (Toyobo, Osaka, Japan) with random hexamers. Primers (Table S5) were designed using Primer Premier 5.0 (Premier Biosoft, Palo Alto, USA). PCR was conducted in a 20 μL reaction system consisting of 10 μL 2 × SYBR Green PCR Master Mix (Toyobo), 200 nM primers, and 2 μL of 1:40-diluted cDNA using the DNA Engine Option 2 Real-Time PCR Detection System and Opticon Monitor software (Bio-Rad, USA). *MaACT*1 was selected as a housekeeping gene. The relative expressions of target genes were determined by the 2^-△△^Ct method [70]. The primers used for qRT-PCR were listed in Table S5.

### Phylogenetic tree construction of MAPK

MAPK genes were isolated from the genome database of Musa (http://banana- genome-hub.southgreen.fr/home) based on annotation and BLAST [71]. Firstly, the sequences belonging to MAPK were downloaded. Then, a further batch of related sequences was obtained from the genome database using *Arabidopsis thaliana* MAPK genes and the BLAST algorithm (TBLASTN and BLASTP). All of the sequences with the highest similarity were chosen as the candidates. Non-redundant banana MAPK sequences and *Arabidopsis thaliana* homologs were aligned using the ClustalX program and named according to the phylogenetic tree, which was constructed with FigTree v1.3.1. The deduced amino acid sequences of *Arabidopsis thaliana* MAPKs were obtained from The *Arabidopsis* Information Resource (TAIR).

### Southern blotting analysis

Total genomic DNA was isolated from young leaf tissues (3.0 g) of MaMAPK3 RNAi transgenic Dajiao plants and Dajiao WT plants using plant DNA isolation kit (TAKARA, Japan). Total genomic DNA was isolated from young leaf tissues (3.0 g) of MaICE1-overexpressing plants and Cavendish banana WT plants using plant DNA isolation kit (TAKARA, Japan). Purified DNA was digested with EcoR V and fractionated on 1.0% agarose gel. The DNA bands were then transferred onto a nylon membrane (Amersham, USA) by upward capillary in a 20× SSC buffer as previously described [72, 73]. The HPT DNA was adopted as a probe, and it was prepared from Hind III restricted fragment of pYL-RNAi (Clontech) and labeled with digoxigenin. Labeling, hybridization, and washing were carried out using the DIG labeling and Luminescent Detection Kits (Roche, Switzerland).

### Yeast two-hybrids assays

The Gal-4 reporter-based ProQuest™ two-hybrid system (Invitrogen, Darmstadt, Germany) was employed to identify the interactions between MAPKs and MAPKKs. Coding sequences (CDSs) of all the MAPK members of Musa were cloned into the prey vector (pDEST32), and CDSs of MAPKKs were introduced into the bait vector (pDEST22). All the combinations of each bait and prey plasmid were PEG-transformed into the yeast strain MaV203 following the protocol of the ProQuest™ two-hybrid system. Positive transformants were first chosen in the synthetic dropout (SD) medium without leucine and tryptophan (SD/−Leu/−Trp), and the culture was transferred to the selection medium without leucine, tryptophan, histidine, and adenine. 3-Amino-1,2,4-triazole (3-AT) was supplemented to the selection plates to inhibit the auto-activation of the prey vectors.

### BiFC assay

The full-length MaPOD P7 and MaICE1 homolog open reading frame was amplified with the following primers set: forward (5′-ggtgagctcggtaccaagctt ATGGCCACCTCCTGGAGAAGCTG-3′)/reverse (5′-agcggccgcactagtaagctt GTTCA CCTTCCTGCAATCCAACCT-3′) and forward (5′- ggtgagctcggtaccaagctt ATGCTCTCGGGGATCAATGG-3′)/reverse (5′-agcggccgcacta-gtaagcttTGACACT GTATTATCGAAGCCGG-3′) respectively, and then introduced into the pMD18-T vector for sequencing. The right MaPOD P7 open reading frame fragment was collected and subcloned into the pRTVnVC vector containing a red fluorescent protein (mCherry) reporter gene (digested with Hind III in advance) to produce the fusion construct Ubi: POD-mVenusC under control of the Ubi promoter by using a One Step Cloning Kit (Vazyme Biotech, Nanjing, China). In the same way, the right MaICE1 open reading frame fragment was collected and subcloned into the pRTVnVN vector containing CFP protein reporter gene to produce the fusion construct Ubi: ICE1-mVenusN. The BiFC system used in this study was as described previously with slight modifications [74]. For the interaction studies, protoplasts (100 μl) (1.5–2 × 106 cells) were transformed with 5–10 μg of plasmids (Ubi:ICE1-mVenusN + Ubi:POD-mVenusC) by the polyethylene glycol (PEG) method with minor modifications [75]. The protoplasts were incubated at 30 °C for 15 h. The localization or co-localization of mVenus proteins and their markers was assessed with a confocal microscope (Olympus BX61, Tokyo, Japan). The full-length CDS of MaMAPK3 was introduced into pRTVnVC vector, and the full-length CDS of *MaICE1* was cloned into pRTVnVN vector. Protoplast isolation and transient expression were carried out as previously described. Empty vectors were co-transformed as negative controls.

### Statistical analysis

A completely randomized design (CRD) was used in the present study. The data were expressed as means ± standard error (SE). Statistical analysis was carried out using ANOVA by DPS software (version 3.01; Zhejiang University, Hangzhou, China). *P* < 0.05 was considered statistically significant.

### Limitations

Limitations include the lack of three independent transgenic lines for gene function analysis and transgenic analysis. In our research, all the transgenic lines showed the same phenotype, so we selected two lines with the most representative and single copy for subsequent experiments such as gene expression and physiological data determination. Three biological duplications were used for each treatment, which made the research results have good repeatability and reliability.

## Supplementary Information

**Additional file 1 Fig. S1**. (A) Phylogenetic tree of MAPKs from *Musa acuminate* and *Arabidopsis.* (B) The expressions of *MAPK* family in Cavendish banana and ‘Dajiao’ under 3 h cold stress. **Fig. S2**. Subcellular localization analysis of MaMAPK3. The ORF of MaMAPK3 was in frame with the GFP C-terminus. (A) Schematic diagrams of the construct used for the subcellular localization assay. (B) Subcellular localization of MaMAPK3 in Cavendish banana protoplast (bar:10 μm). **Fig. S3**. Browning phenomenon of *MAMAPK3*-overexpressing resistant embryogenic calli. **Fig. S4**. PCR analysis of MaMAPK3 RNAi transgenic ‘Dajiao’ lines (M: DNA molecular weight marker; P: plasmid DNA). **Fig. S5**. Southern blotting analysis of transformed MaMAPK3 RNAi transgenic ‘Dajiao’ plants. M: DNA molecular weight marker; P: plasmid DNA; WT: wild type. **Fig. S6**. Subcellular localization analysis of MaICE1. The ORF of MaICE1 was in frame with the GFP C-terminus. (A) Schematic diagrams of the construct used for the subcellular localization assay. (B) Subcellular localization of MaICE1 in Cavendish banana protoplast (bar:10 μm). **Fig. S7**. Generation and molecular identification of transgenic banana plants overexpressing MaICE1. PCR confirmation of the hygromycin-resistant plants using (A) hpt-specific primers or (B) Pubi-MaICE1 primers. M, DNA molecular weight marker; WT, wild-type; −, water; the numbers indicate different transgenic lines (lines 1, 5, 11, and 13 are designated as #1, #5, #11, and #13, respectively); P, plasmid DNA (used as a positive control). (C) Southern blot analysis of *MaICE1*-overexpressing transgenic ‘Cavendish banana’ lines. M: DNA Molecular- Weight Marker; P: plasmid DNA; WT: wild type. (D) Expression analysis of *MaICE1* in four transgenic lines by RT-PCR. The *MaACT1* gene was used as an internal control. **Fig. S8**. Heatmap of physical interaction verified by Y2H assay. (A) Physical interaction between MaICE1 and MaMAPKs. (B) Physical interaction between MaMAPKs and MaMKKs. **Fig. S9**. Heatmap of physical interaction between MaMAPKs and MaMKKs verified by Y2H assay. **Fig. S10**. The plasmid maps of all the constructs used for transgenic in the study.

**Additional file 2 Table S1**. Transcriptomic analysis between WT and Ox-ICE1 (line13) under 0 h cold treatment.

**Additional file 3 Table S2**. Transcriptomic analysis between WT and line13 under 1 h cold treatment.

**Additional file 4 Table S3**. Transcriptomic analysis between WT and line13 under 4 h cold treatment.

**Additional file 5 Table S4**. The expression pattern of all identified MAPK cascade genes in the transcriptome.

**Additional file 6 Table S5**.

## Data Availability

The sequencing raw data of this article have been deposited in a SRA database at the NCBI -https://www.ncbi.nlm.nih.gov/Traces/study/?acc=PRJNA439180.

## Abbreviations

WT: wild type
MAPKKK: mitogen-activated protein kinase kinase kinase
MAPKK: mitogen-activated protein kinase kinase
MAPK: mitogen-activated protein kinase
bHLH TF: basic-helix-loop-helix transcription factor
qRT-PCR: quantitative real-time PCR
GFP: green fluorescent protein
ORF: open reading frame
MDA: malondialdehyde
Pro: proline
DEGs: differentially expressed genes
*POD P7*: *Peroxidase P7*
DAB: 3,3′-diaminobenzidine
NBT: Nitroblue Tetrazolium
CaMV 35S: cauliflower mosaic virus 35S promoter
dsRNA: double-stranded RNA interference
ECSs: suspension cultured cells
CDSs: coding sequences
3-AT: 3-Amino-1,2,4-triazole
PEG: polyethylene glycol
CRD: completely randomized design
SE: standard error
