# Supplementary material for: MaMAPK3-MaICE1-MaPOD P7 pathway, a positive regulator of cold tolerance in banana
Source: BMC Plant Biol. 2021 Feb 17;21:97. doi: 10.1186/s12870-021-02868-z (PMC7890976; doi:10.1186/s12870-021-02868-z)
Supplement: Supplementary file 1 — Additional file 1 Fig. S1. (A) Phylogenetic tree of MAPKs from Musa acuminate and Arabidopsis. (B) The expressions of MAPK family in Cavendish banana and ‘Dajiao’ under 3 h cold stress. Fig. S2. Subcellular localization analysis of MaMAPK3. The ORF of MaMAPK3 was in frame with the GFP C-terminus. (A) Schematic diagrams of the construct used for the subcellular localization assay. (B) Subcellular localization of MaMAPK3 in Cavendish banana protoplast (bar:10 μm). Fig. S3. Browning phenomenon of MAMAPK3-overexpressing resistant embryogenic calli. Fig. S4. PCR analysis of MaMAPK3 RNAi transgenic ‘Dajiao’ lines (M: DNA molecular weight marker; P: plasmid DNA). Fig. S5. Southern blotting analysis of transformed MaMAPK3 RNAi transgenic ‘Dajiao’ plants. M: DNA molecular weight marker; P: plasmid DNA; WT: wild type. Fig. S6. Subcellular localization analysis of MaICE1. The ORF of MaICE1 was in frame with the GFP C-terminus. (A) Schematic diagrams of the construct used for the subcellular localization assay. (B) Subcellular localization of MaICE1 in Cavendish banana protoplast (bar:10 μm). Fig. S7. Generation and molecular identification of transgenic banana plants overexpressing MaICE1. PCR confirmation of the hygromycin-resistant plants using (A) hpt-specific primers or (B) Pubi-MaICE1 primers. M, DNA molecular weight marker; WT, wild-type; −, water; the numbers indicate different transgenic lines (lines 1, 5, 11, and 13 are designated as #1, #5, #11, and #13, respectively); P, plasmid DNA (used as a positive control). (C) Southern blot analysis of MaICE1-overexpressing transgenic ‘Cavendish banana’ lines. M: DNA Molecular- Weight Marker; P: plasmid DNA; WT: wild type. (D) Expression analysis of MaICE1 in four transgenic lines by RT-PCR. The MaACT1 gene was used as an internal control. Fig. S8. Heatmap of physical interaction verified by Y2H assay. (A) Physical interaction between MaICE1 and MaMAPKs. (B) Physical interaction between MaMAPKs and MaMKKs. Fig. S9. Heatmap of [file 12870_2021_2868_MOESM1_ESM.pptx]

## Slide 1
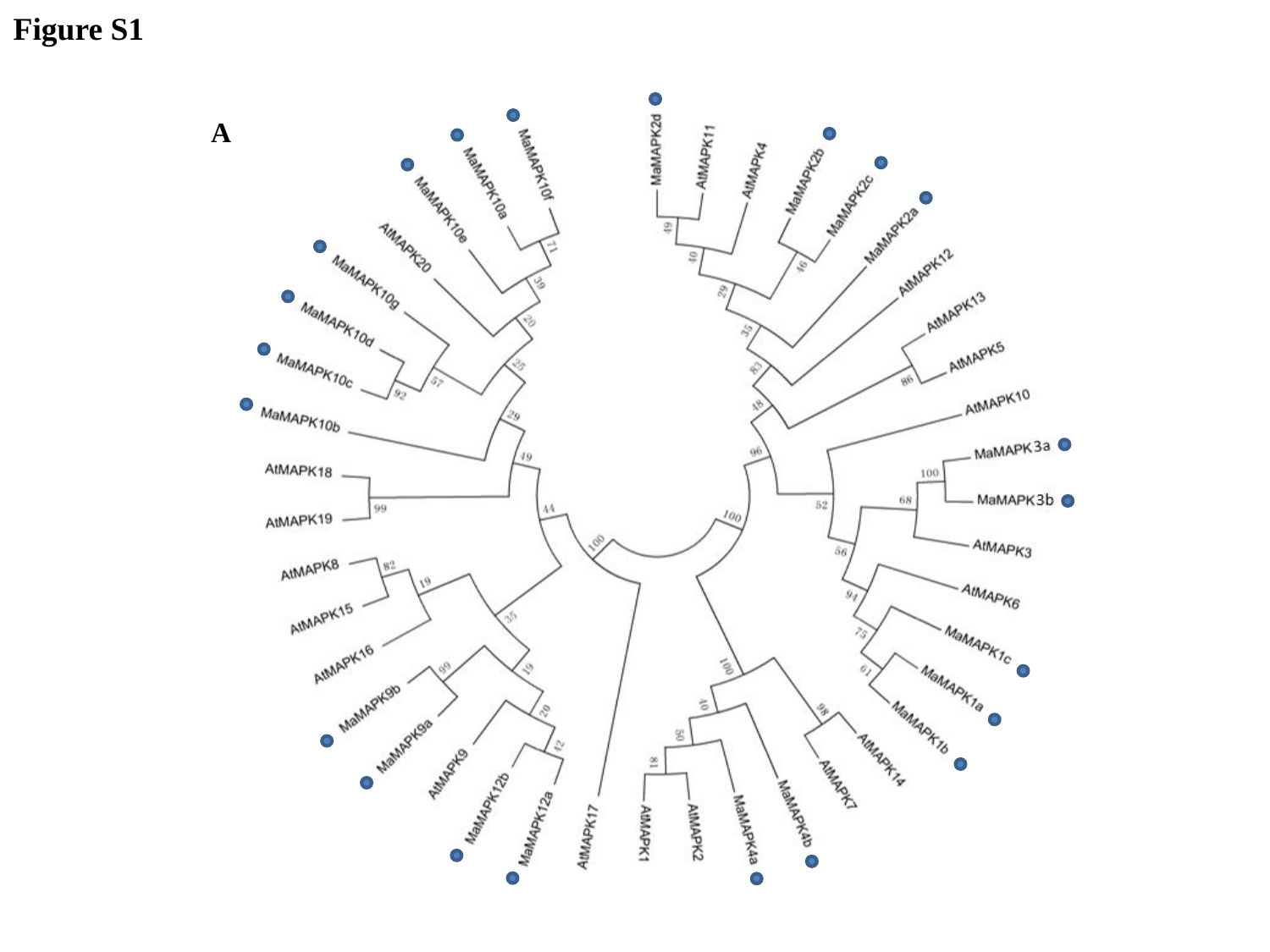

Figure S1
A

## Slide 2
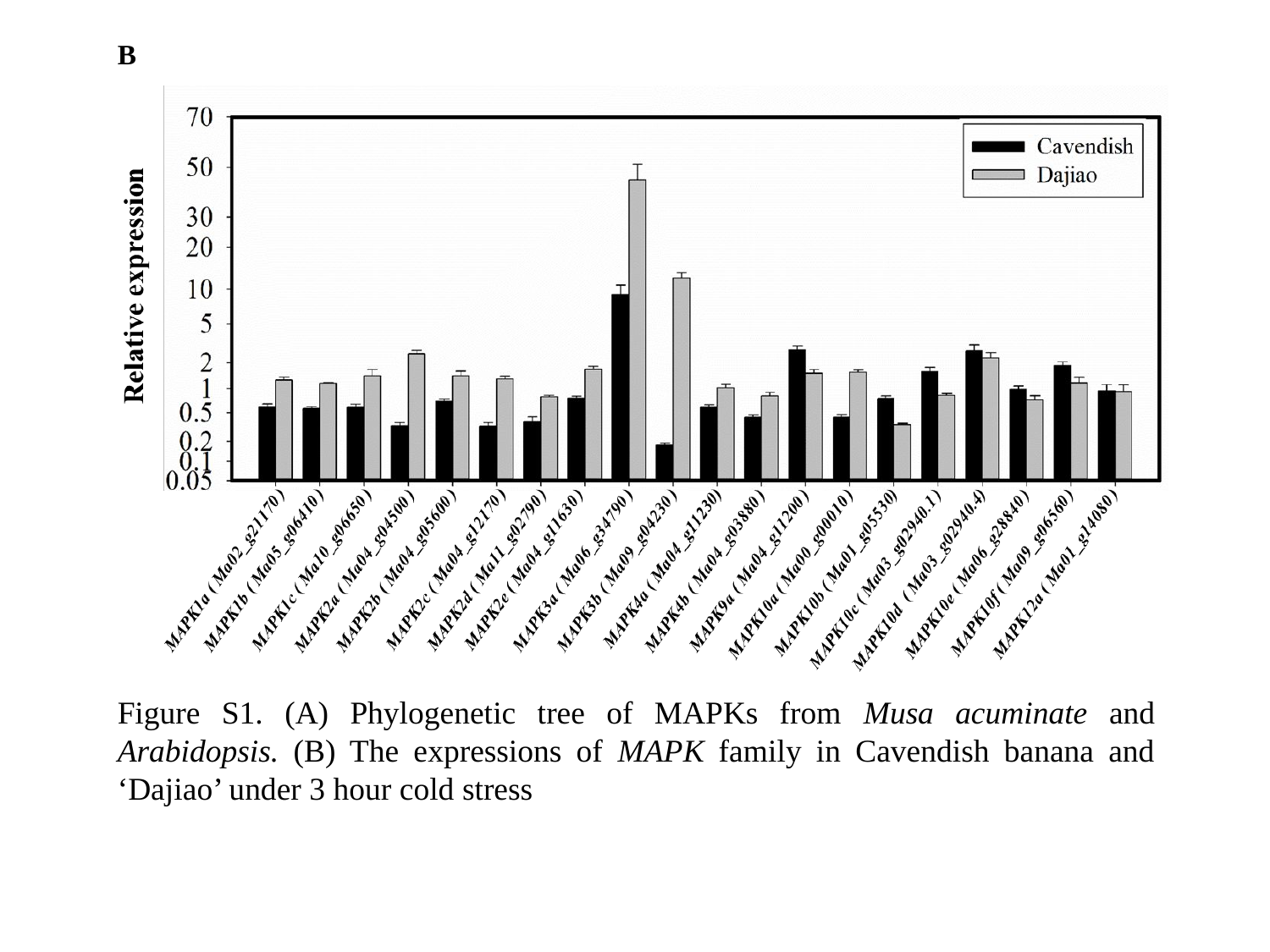

B
Figure S1. (A) Phylogenetic tree of MAPKs from Musa acuminate and Arabidopsis. (B) The expressions of MAPK family in Cavendish banana and ‘Dajiao’ under 3 hour cold stress

## Slide 3
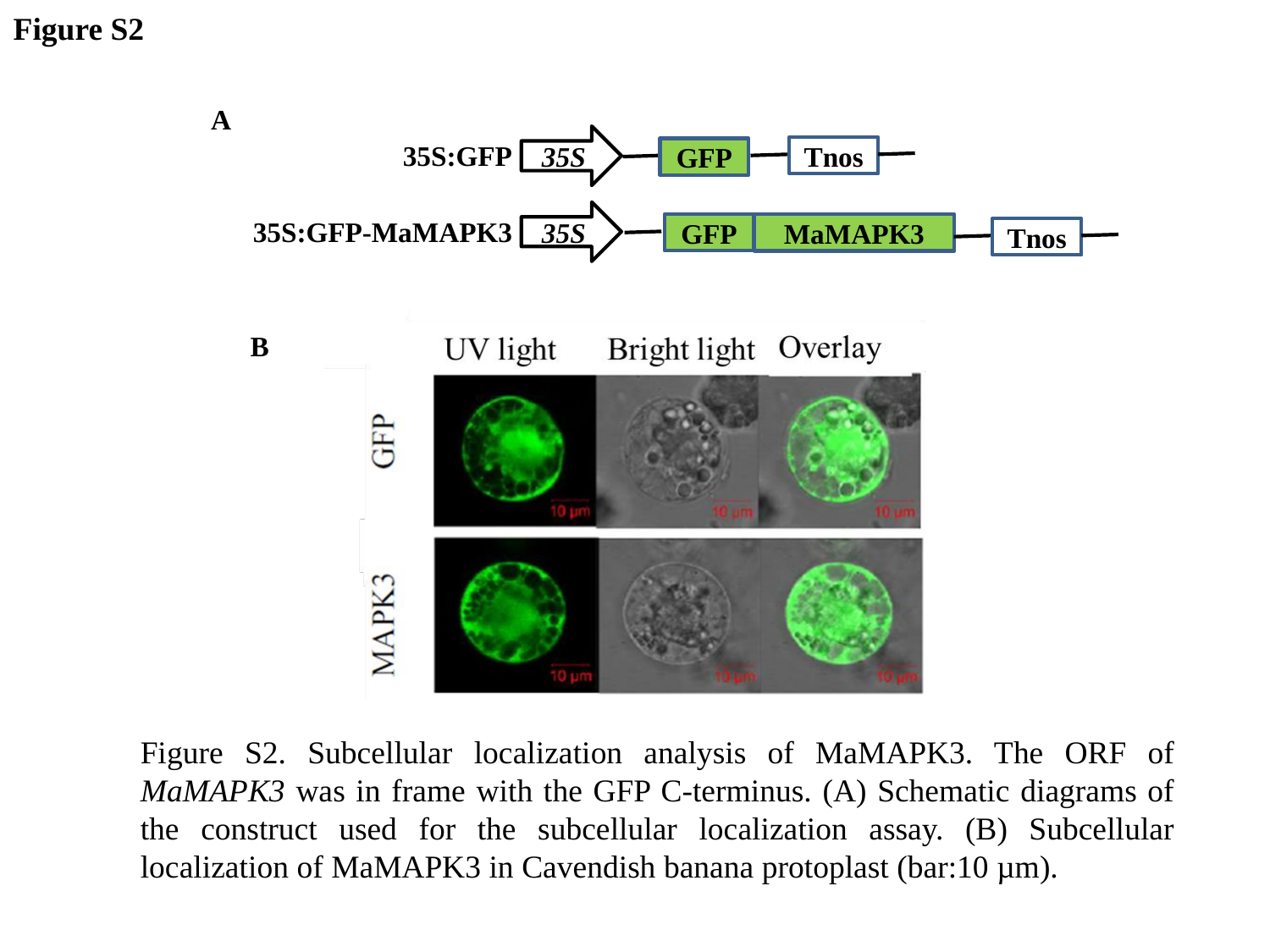

Figure S2
A
35S
35S:GFP
Tnos
GFP
35S
35S:GFP-MaMAPK3
GFP
MaMAPK3
Tnos
B
Figure S2. Subcellular localization analysis of MaMAPK3. The ORF of MaMAPK3 was in frame with the GFP C-terminus. (A) Schematic diagrams of the construct used for the subcellular localization assay. (B) Subcellular localization of MaMAPK3 in Cavendish banana protoplast (bar:10 µm).

## Slide 4
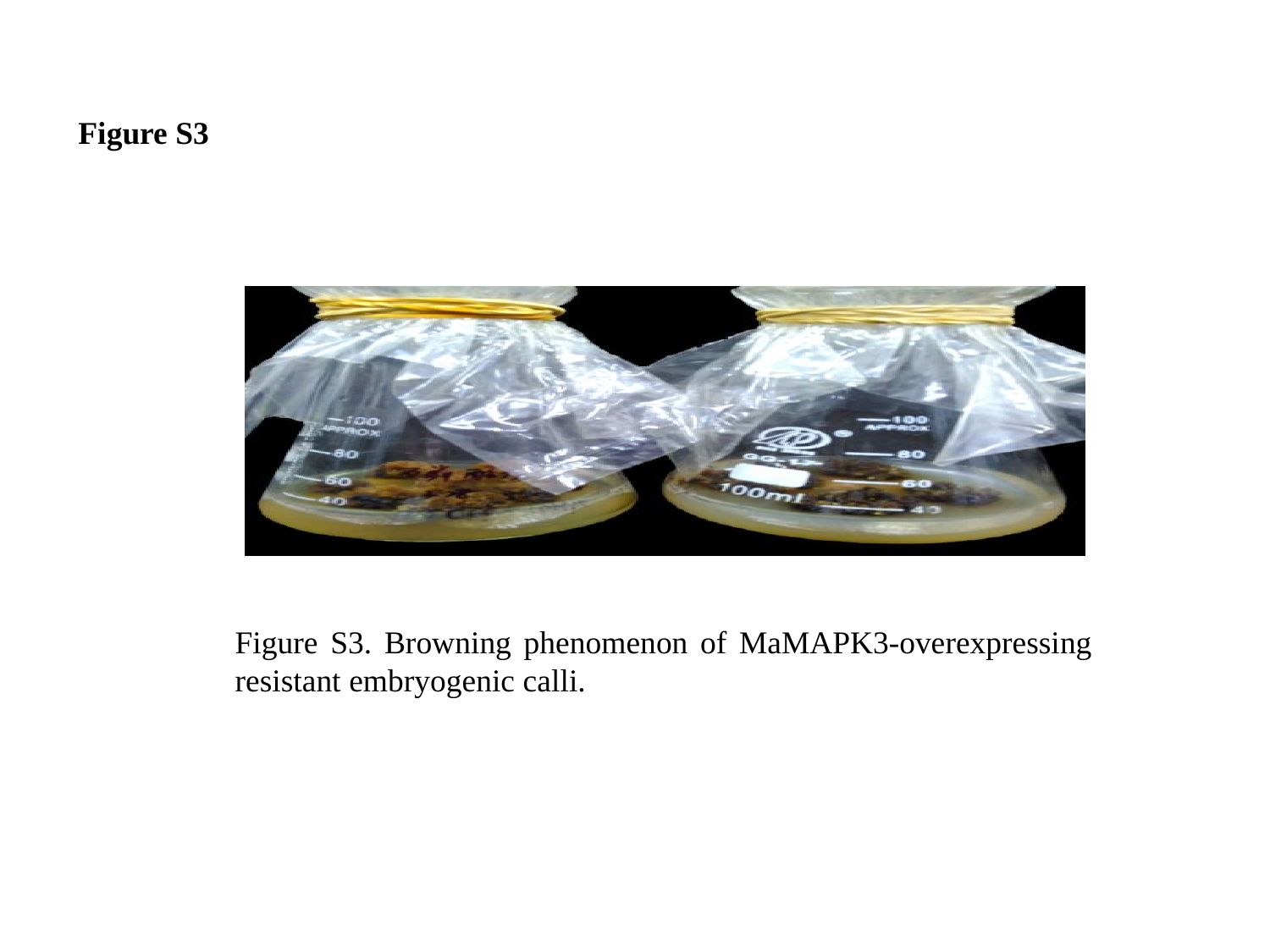

Figure S3
Figure S3. Browning phenomenon of MaMAPK3-overexpressing resistant embryogenic calli.

## Slide 5
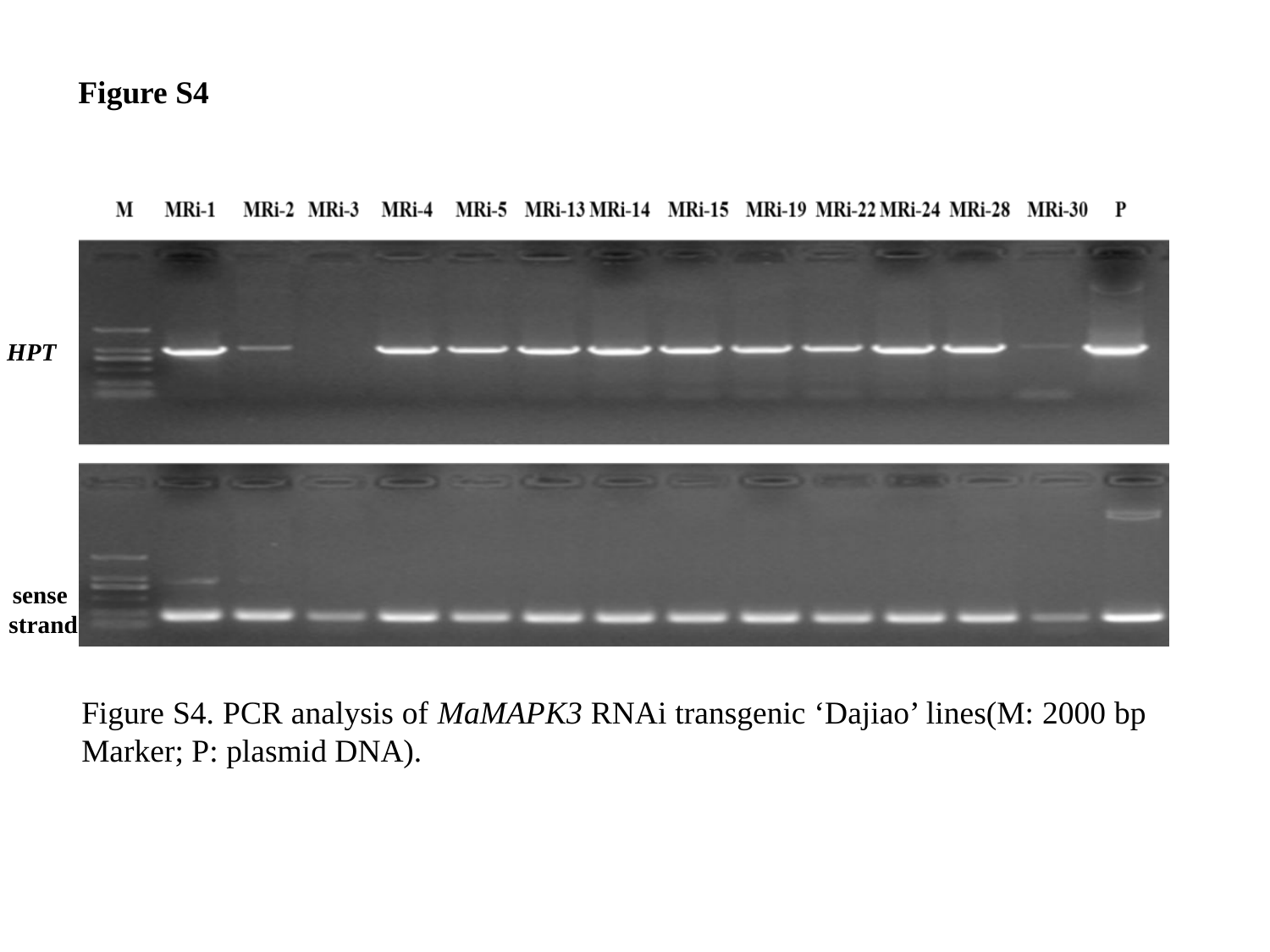

Figure S4
HPT
sense
strand
Figure S4. PCR analysis of MaMAPK3 RNAi transgenic ‘Dajiao’ lines(M: 2000 bp Marker; P: plasmid DNA).

## Slide 6
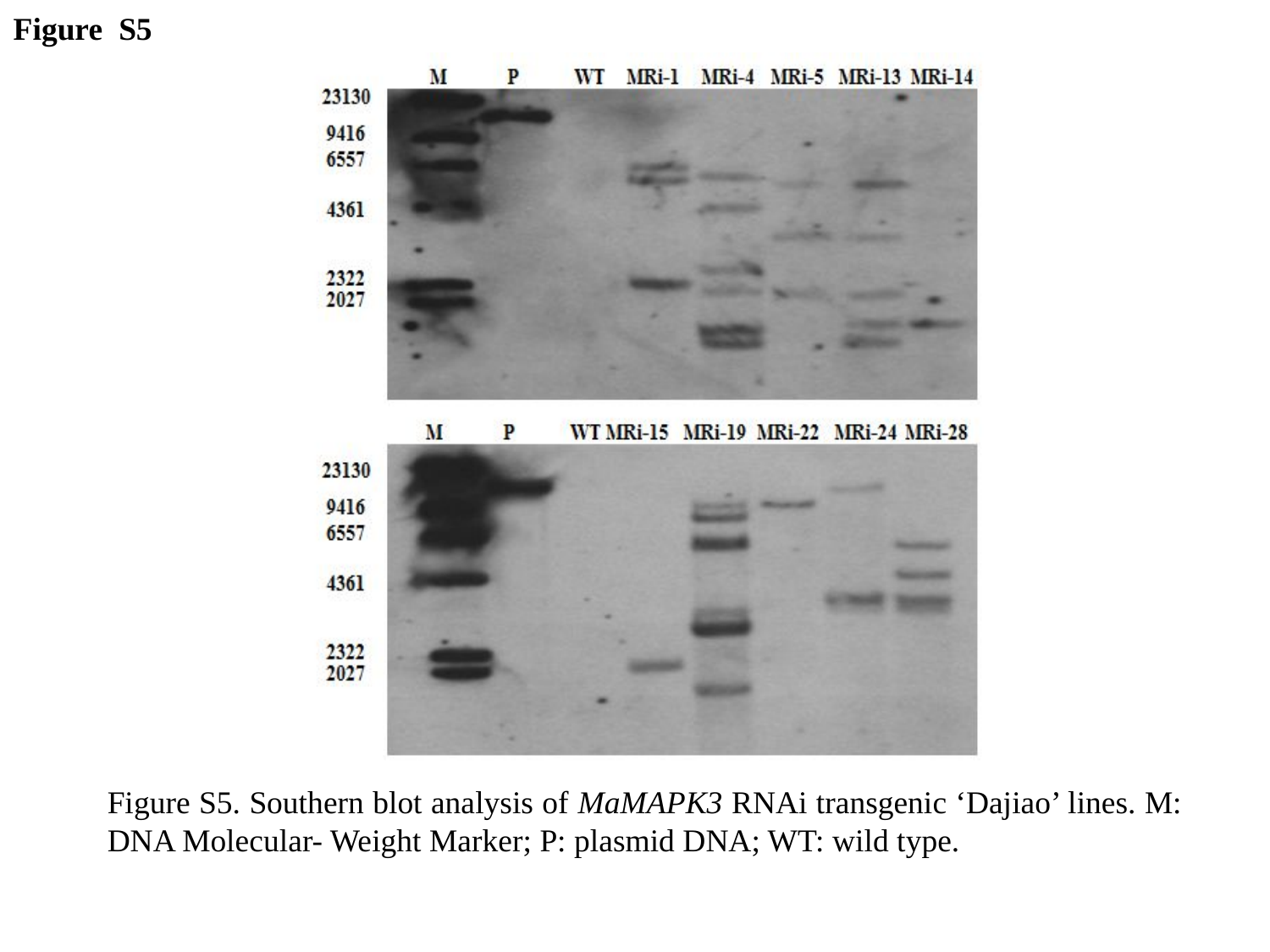

Figure S5
Figure S5. Southern blot analysis of MaMAPK3 RNAi transgenic ‘Dajiao’ lines. M: DNA Molecular- Weight Marker; P: plasmid DNA; WT: wild type.

## Slide 7
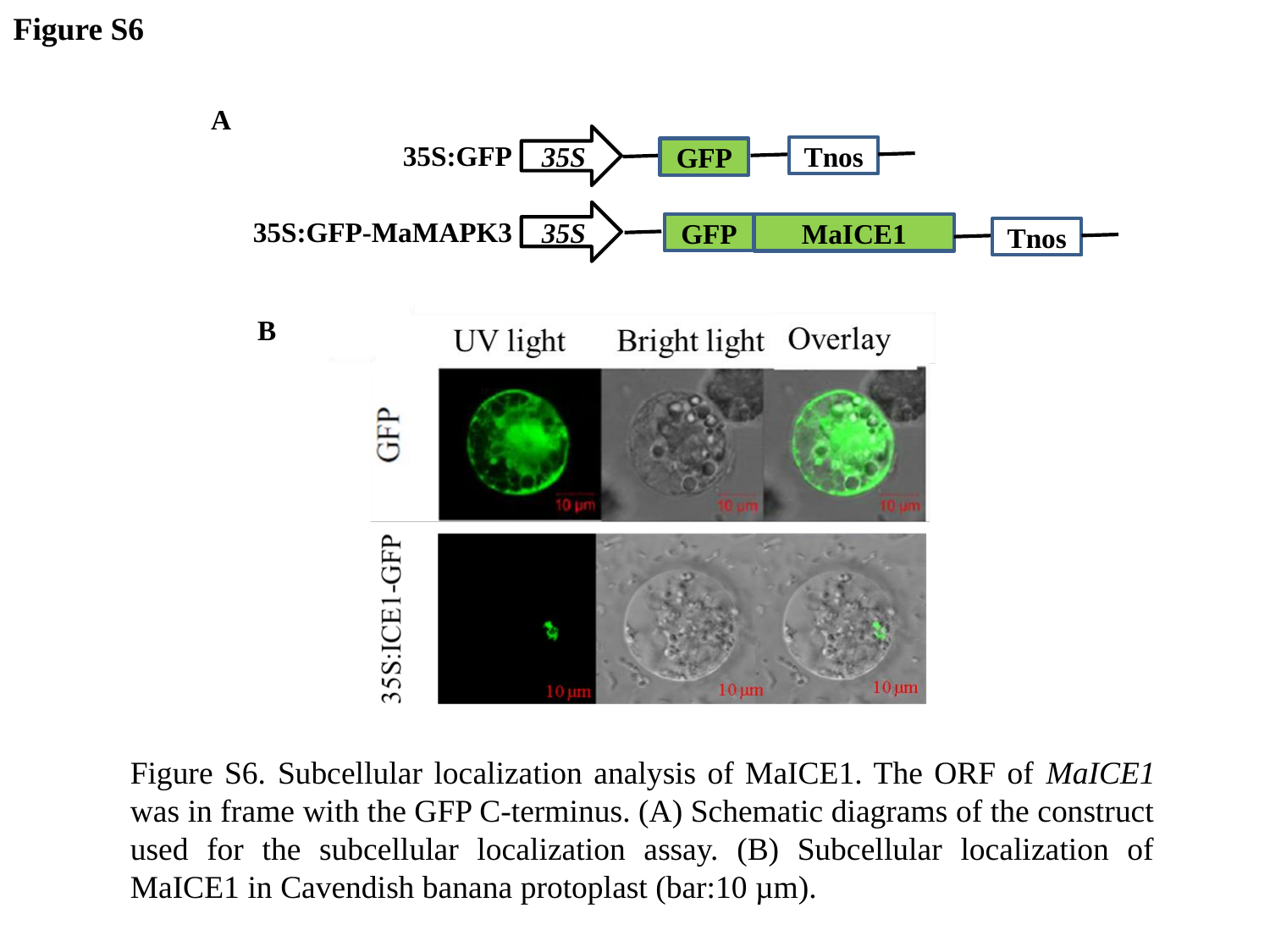

Figure S6
A
35S
35S:GFP
Tnos
GFP
35S
35S:GFP-MaMAPK3
GFP
MaICE1
Tnos
B
Figure S6. Subcellular localization analysis of MaICE1. The ORF of MaICE1 was in frame with the GFP C-terminus. (A) Schematic diagrams of the construct used for the subcellular localization assay. (B) Subcellular localization of MaICE1 in Cavendish banana protoplast (bar:10 µm).

## Slide 8
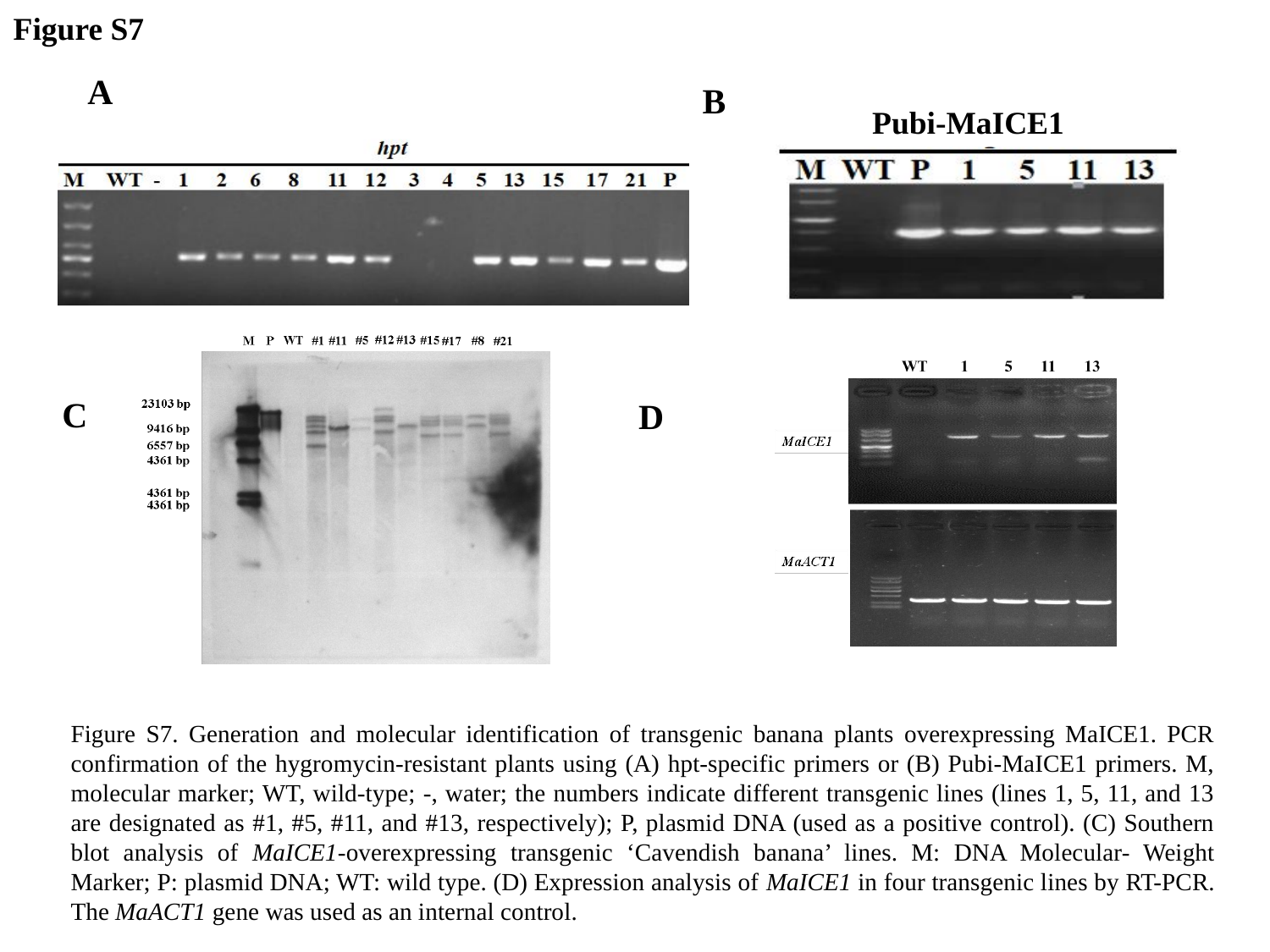

Figure S7
A
B
Pubi-MaICE1
C
D
Figure S7. Generation and molecular identification of transgenic banana plants overexpressing MaICE1. PCR confirmation of the hygromycin-resistant plants using (A) hpt-specific primers or (B) Pubi-MaICE1 primers. M, molecular marker; WT, wild-type; -, water; the numbers indicate different transgenic lines (lines 1, 5, 11, and 13 are designated as #1, #5, #11, and #13, respectively); P, plasmid DNA (used as a positive control). (C) Southern blot analysis of MaICE1-overexpressing transgenic ‘Cavendish banana’ lines. M: DNA Molecular- Weight Marker; P: plasmid DNA; WT: wild type. (D) Expression analysis of MaICE1 in four transgenic lines by RT-PCR. The MaACT1 gene was used as an internal control.

## Slide 9
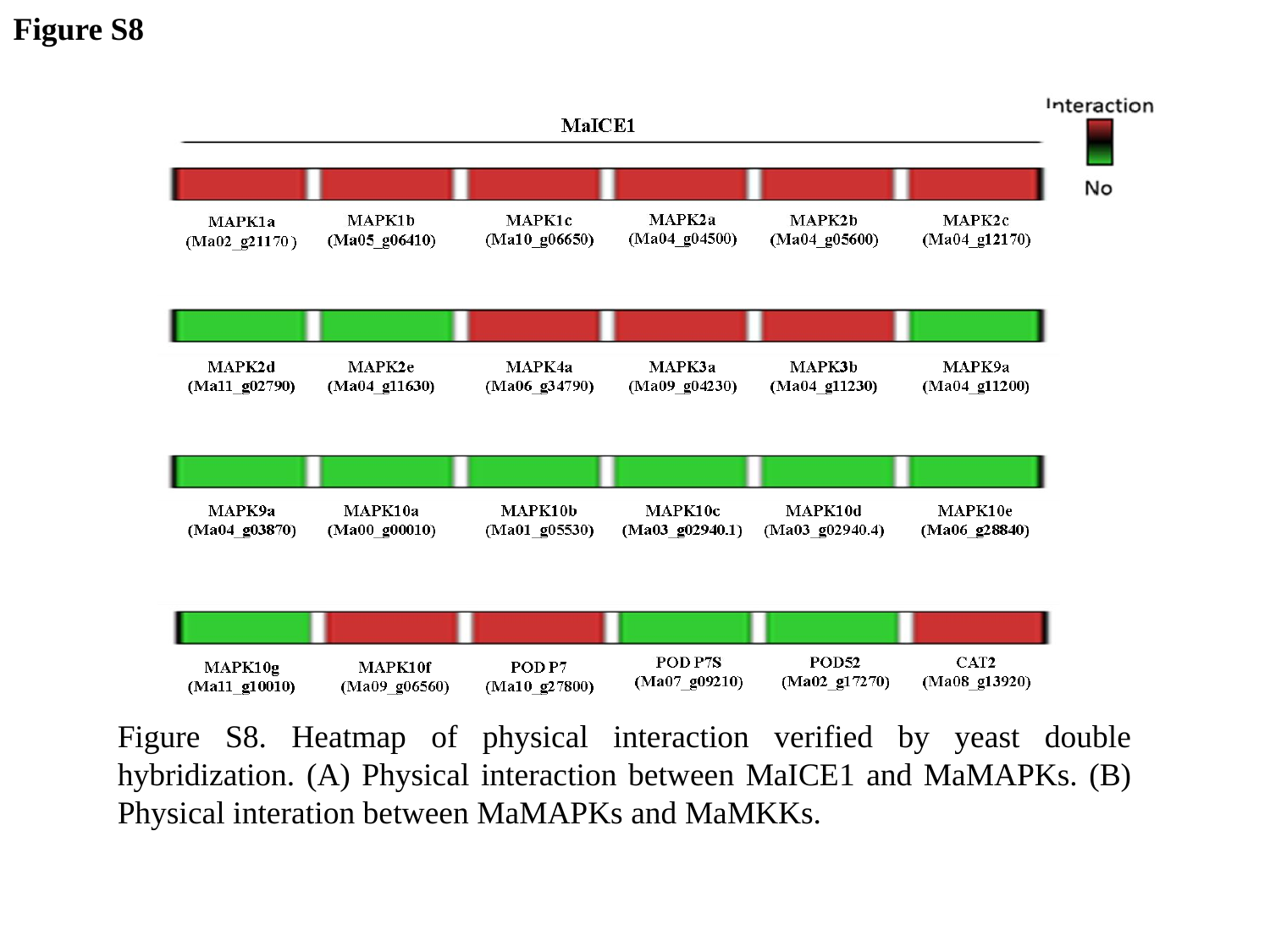

Figure S8
Figure S8. Heatmap of physical interaction verified by yeast double hybridization. (A) Physical interaction between MaICE1 and MaMAPKs. (B) Physical interation between MaMAPKs and MaMKKs.

## Slide 10
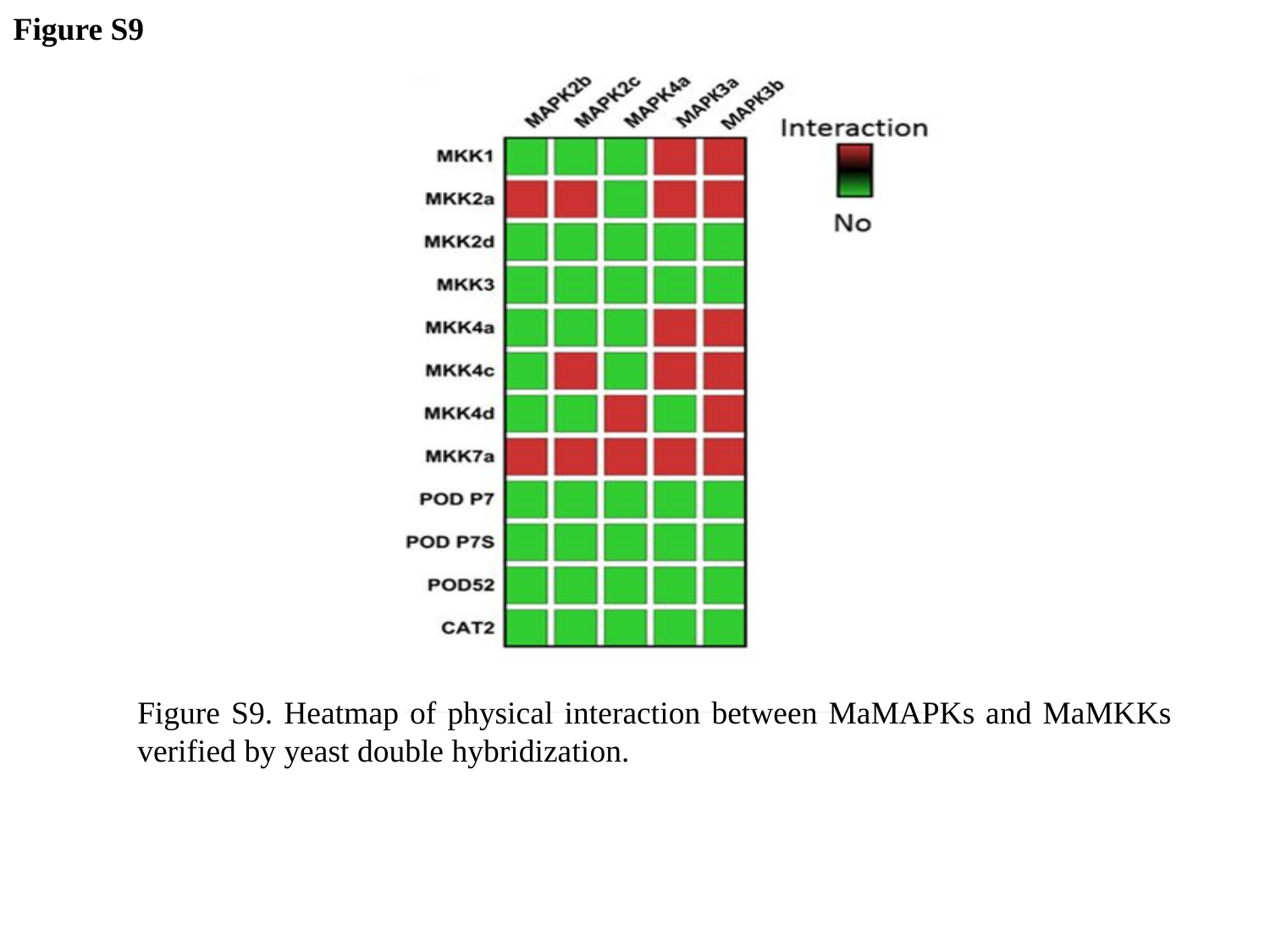

Figure S9
Figure S9. Heatmap of physical interaction between MaMAPKs and MaMKKs verified by yeast double hybridization.

## Slide 11
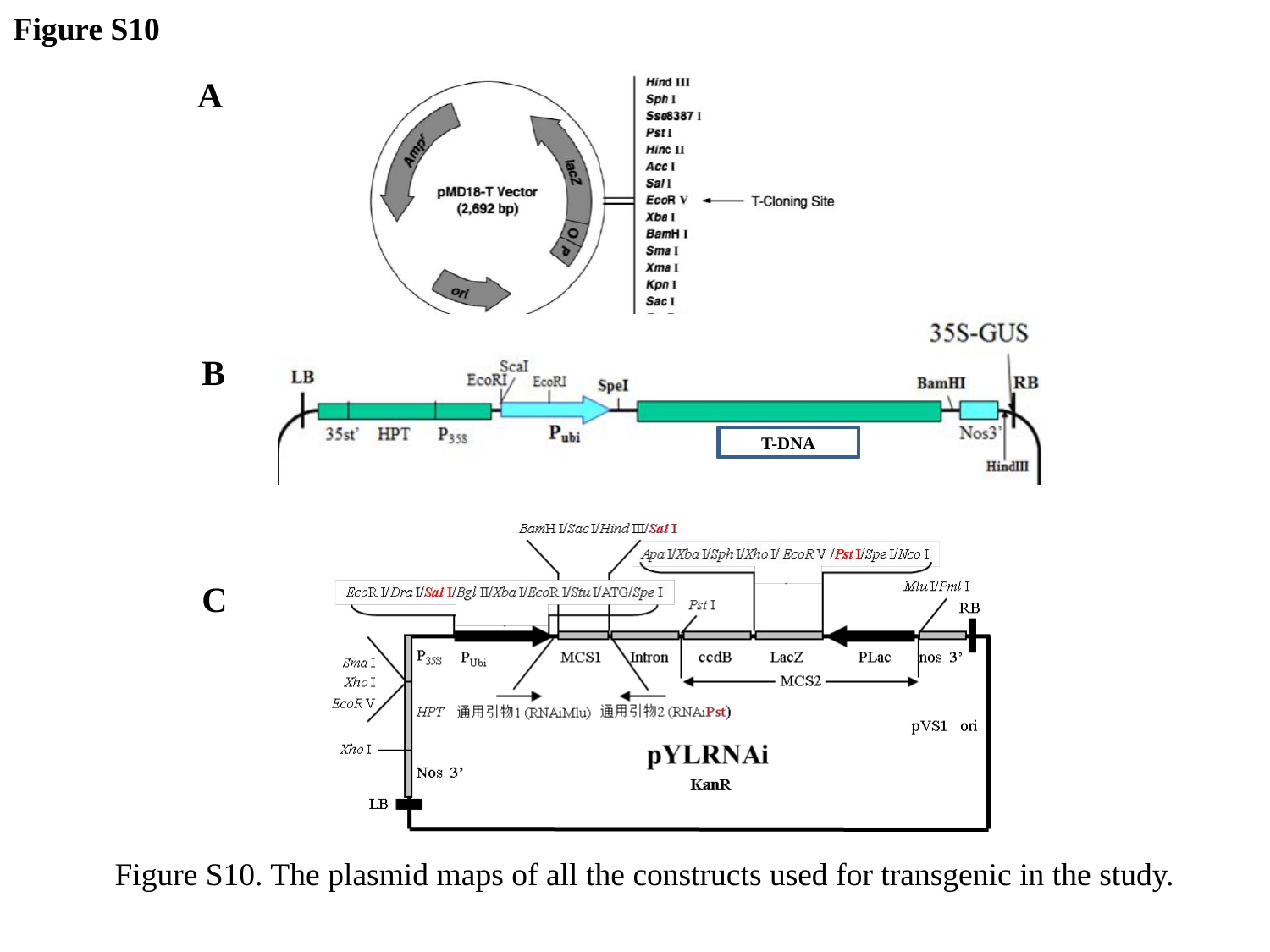

Figure S10
A
T-DNA
B
C
Figure S10. The plasmid maps of all the constructs used for transgenic in the study.
